# Total synthesis of (+)-grandiamide D, dasyclamide and gigantamide A from a Baylis–Hillman adduct: A unified biomimetic approach

**DOI:** 10.3762/bjoc.10.9

**Published:** 2014-01-10

**Authors:** Andivelu Ilangovan, Shanmugasundar Saravanakumar

**Affiliations:** 1 School of Chemistry, Bharathidasan University, Tiruchirappalli, 620024, India; 2 Syngene International Ltd., Bangalore, 560 099, India

**Keywords:** Baylis–Hillman reaction, dasyclamide, gigantamide A, (+)-grandiamide D, natural products, putrescine bisamides

## Abstract

A unified strategy was followed for the synthesis of three putrescine bisamides, (+)-grandiamide D, dasyclamide and gigantamide A, isolated from leaves of *Aglaia gigantea,* by making use of a common synthetic intermediate prepared by the Baylis–Hillman reaction. Asymmetric synthesis of the natural (+)-grandiamide D was accomplished from camphor sultam.

## Introduction

**Figure 1:**
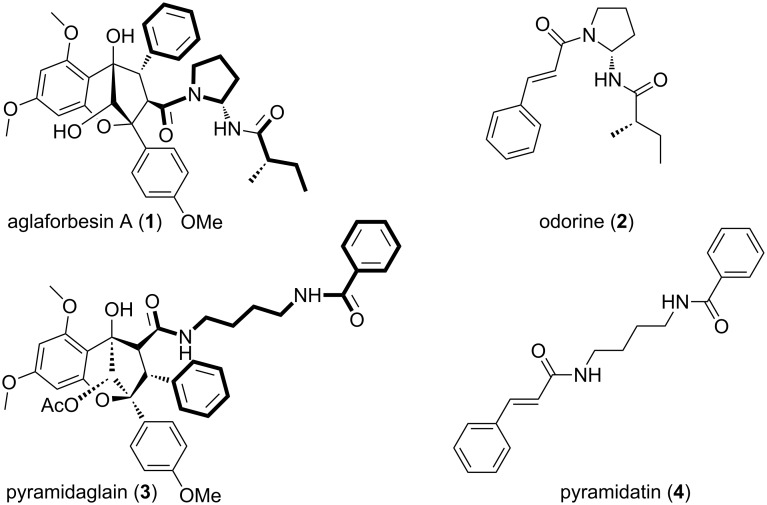
Bisamides as building blocks for flavaglins.

Putrescine bisamides are one of the important naturally occurring polyamine alkaloids found in open chain and cyclic forms. The genera *Aglaia* are the richest source of putrescine bisamides. The cyclic 2-aminopyrrolidine compounds are synthesised in plants from open-chained bisamides by enzymatic cyclization. It is assumed that both forms of bisamides undergo a cycloaddition reaction with a co-occurring flavanol to form highly bioactive flavaglines [1–2]. For example, odorine (**2**) acted as a building block for aglaforbesin A (**1**) and pyramidatin (**4**) for pyramidaglain (**3**) as shown in Figure 1 [3]. The benzopyran and benzoxepine containing flavaglins showed insecticidal, antifungal and antiproliferative activity against various cancer cell lines [4]. Apart from acting as precursors for flavaglins, some of the cyclic bisamides such as odorine, dehydroodorine and odorinol exhibited cytotoxic and antiviral activity [5].

There is only a limited number of putrescine bisamides isolated from natural sources and the amount of synthetic approaches known for these structurally interesting and biologically important molecules is quite low. Except for the elaborate synthetic work carried out by Hesse and Detterbeck [6], who described the synthesis of nine putrescine bisamides isolated from different *Aglaia* species along with the structural revision for one of the compounds and determination of absolute configuration for another compound, no other literature precedence is available till now.

**Figure 2:**
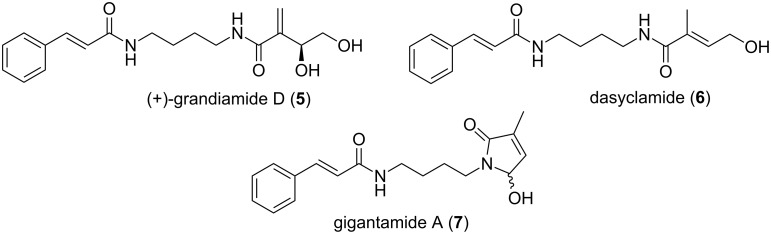
(+)-Grandiamide D, gigantamide A and dasyclamide.

Duong et al. have isolated three cinnamoyl bisamide derivatives, two new compounds grandiamide D, gigantamide A and the known compound dasyclamide (Figure 2) from the leaves of *Aglaia gigantea* [7]. Even though the structures were determined by spectroscopic means, the total synthesis of these molecules has not been explored so far. As a result of their co-existence, we assumed that these compounds might have been formed from a single precursor with the help of elements present in the plant. Hence we decided to adopt a unified synthetic strategy for the synthesis of all the three compounds **5–7** by mimicking the biosynthetic pathway.

Baylis–Hillman adducts are densely functionalized synthons, which mainly consist of three different functional groups such as the ester, olefin and hydroxy group [8]. Recent developments made it possible to introduce additional functional groups on them by replacing the starting materials by activated alkenes and aldehydes. Various multifunctional molecules including natural products have already been successfully synthesized using the Baylis–Hillman adducts [9].

**Scheme 1:**
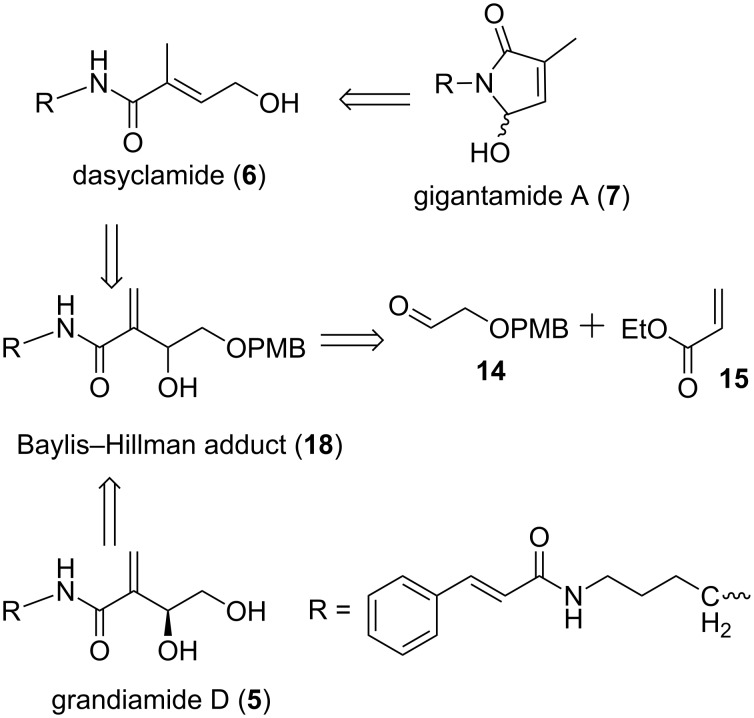
Retrosynthetic analysis: A unified synthetic approach for the synthesis of grandiamide D, dasyclamide and gigantamide A.

Structural features of grandiamide D, gigantamide A, dasyclamide reveal that the *N*-(4-aminobutyl)cinnamamide segment is common and a remaining part of these molecules could be constructed from this common intermediate. The synthetic versatility of the Baylis–Hillman adducts is a perfect platform for the retrosynthetic plan (Scheme 1). Accordingly, 2-(4-methoxybenzyloxy)acetaldehyde was expected to provide a tetra-functionalized Baylis–Hillman adduct (+)-**16** from which grandiamide D can be obtained. A rearrangement of this common intermediate should be able to provide dasyclamide, which on oxidation, should give gigantamide A.

## Results and Discussion

**Scheme 2:**
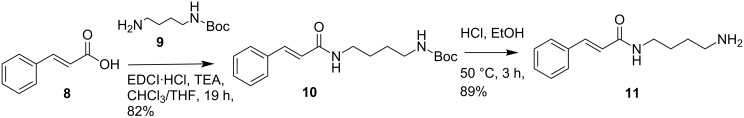
Preparation of *N*-(4-aminobutyl)cinnamamide.

The synthetic plan was started with the preparation of *N*-(4-aminobutyl)cinnamamide (**11**), in two steps from *tert*-butyl 4-aminobutylcarbamate and cinnamic acid by EDCI-mediated coupling followed by removal of the Boc protecting group by refluxing in ethanolic HCl as given in Scheme 2.

### Synthesis of (±)-grandiamide D (**5**)

**Scheme 3:**
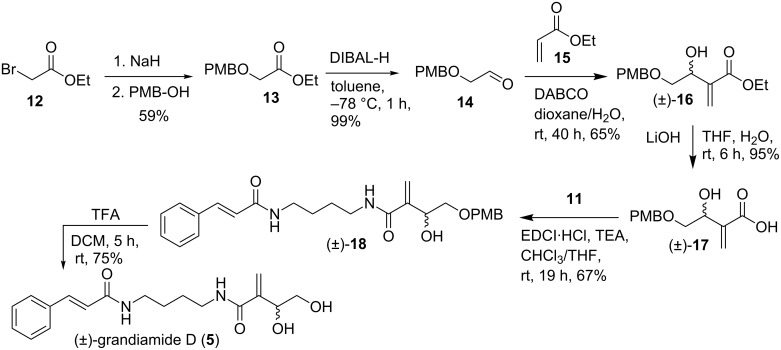
Synthesis of (±)-grandiamide D.

The common and crucial intermediate, β,γ-dihydroxy-α-methylenebutyric acid ((±)**-16**) required a selective protection of the hydroxy group at γ position. The PMB (*p*-methoxybenzyl) group was chosen as a result of its robustness and stability towards a wide range of acidic or basic conditions [10]. The entire pathway leading to the synthesis of (±)-grandiamide D is portrayed in Scheme 3.

The reaction between ethyl bromoacetate (**12**) and *p*-methoxybenzyl alcohol in the presence of NaH (Scheme 3) furnished the desired ester **13** which on reduction with DIBAL-H provided 2-((4-methoxybenzyl)oxy)acetaldehyde (**14**) [11]. In order to reduce the amount of acrylate and to increase the yield of compound (±)-**16**, the Baylis–Hillman reaction between the aldehyde **14** and ethyl acrylate (**15**) was tried using different catalysts such as DBU, quinuclidine [12] and *n*-Bu_3_P. DABCO was found to be a better catalyst and the adduct (±)-**16** was obtained in high yield [13] in dioxane/water (1:1), at room temperature after 40 h [13]. Hydrolysis of the ester (±)-**16** with aqueous LiOH in THF yielded the acid (±)-**17** which on EDCI supported reaction with *N*-(4-aminobutyl)cinnamamide (**11**) gave the PMB protected grandiamide D ((±)-**18**). Among different reagents such as DDQ, CAN, Yb(OTf)_3_ and CeCl_3_–NaI used for deprotection of the PMB group, TFA gave the better result. Even though POCl_3_ [14] was able to effect complete removal of the PMB group in short time, the yield was only moderate. Thus (±)-grandiamide D ((±)-**5**) was obtained in 16.6% overall yield. The spectral data obtained for synthetic compound (±)-**5** matches in all respect with the data reported for the natural product [7].

### Asymmetric synthesis of (+)-grandiamide D (**5**)

**Scheme 4:**
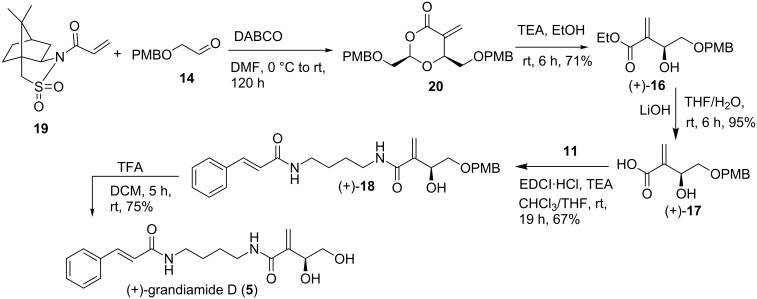
Asymmetric synthesis of natural (+)-grandiamide D.

In continuation, we moved on with an asymmetric approach towards the synthesis of natural product (+)-grandiamide D (**5**). We assumed that the asymmetric Baylis–Hillman reaction [15] would be an ideal way to introduce chirality. Reaction between acryloyl sultam **19**, and 2-((4-methoxybenzyl)oxy)acetaldehyde (**14**) in DMF [12], afforded cyclic product **20**, which was then treated as such with TEA in ethanol to afford enantiopure ester (+)-**16** in 71% yield, after purification (Scheme 4). Chiral HPLC analysis of (**+**)-**16** showed that the enantiomeric ratio is 99.7:0.3 in favour of the (+)-isomer. Further chemistry was carried out as described earlier in Scheme 3, to get (+)-grandiamide D (**5**) in almost the same yield. The enantiomeric purity of (+)-grandiamide D (**5**) was found to be 98.6%, as determined from chiral HPLC analysis. (observed [α]_D_^25^ = +4.7 (*c* 0.5, CHCl_3_); reported [7] [α]_D_^20^ = +2.0 (*c* 0.5, CHCl_3_).

### Synthesis of dasyclamide

**Scheme 5:**
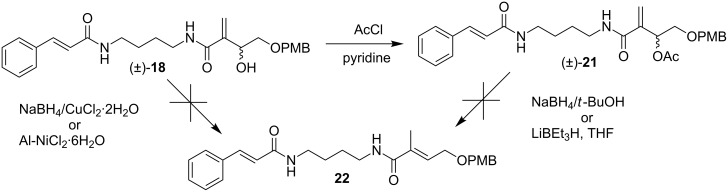
Various approaches for the synthesis of (*E*)-*N*-(4-cinnamamidobutyl)-4-((4-methoxybenzyl)oxy)-2-methylbut-2-enamide (**22**).

Since Baylis–Hillman adduct (±)-**18** was considered as the common intermediate, we decided to start with the same for the synthesis of dasyclamide (**6**). As explained in Scheme 5 attempts to get enamide **22** by reductive dehydroxylation of compound (±)-**18** using NaBH_4_/CuCl_2_·2H_2_O or Al-NiCl_2_·2H_2_O [16–18] and reductive deacetoxylation of compound (±)-**21** using NaBH_4_/*t*-BuOH or LiBEt_3_H, THF [19–21] did not yield fruitful results.

**Scheme 6:**
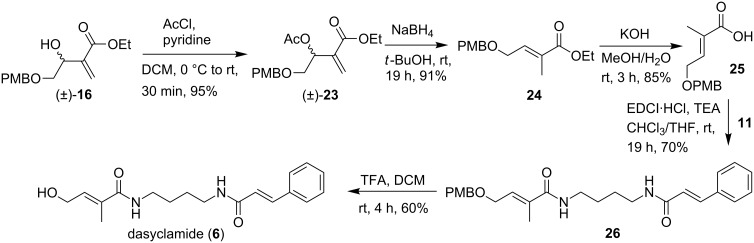
Synthesis of dasyclamide.

These failures pushed us to explore the dehydroxylation or deacetoxylation reaction using a simpler precursor (±)-**16.** However, attempts to dehydroxylate the Baylis–Hillman adduct (±)-**16** with NaBH_4_/CuCl_2_·2H_2_O [17] and Al-NiCl_2_·2H_2_O proved unsuccessful hence, we focused on the deacetoxylation of (±)-**16**. Treatment of (±)-**16** with acetyl chloride in the presence of pyridine afforded the corresponding acetate (±)-**23** in almost quantitative yield, which underwent the desired deacetoxylation with NaBH_4_/*t*-butanol [20] to provide the ester **24** in good yield (Scheme 6). The ester **24** was hydrolyzed with aqueous KOH in methanol at room temperature to get acid **25** which was then treated with *N*-(4-aminobutyl)cinnamamide (**11**) in the presence of EDCI to get enamide **26**. Further, deprotection was carried out with TFA to obtain dasyclamide (**6**) in an overall yield of 9.2% (Scheme 6).

The spectral data is in accordance with the published data for natural dasyclamide [7] which further confirms the structure of the natural product.

### Synthesis of gigantamide A

As given in the retrosynthetic analysis and based on the preference in literature for the preparation of jatropham by photocyclization [22], intramolecular cyclization of the aldehyde **27** was considered for the preparation of gigantamide A (**7**). The aldehyde **27** in turn could be prepared from dasyclamide (**6**). Conditions and results for the oxidation of dasyclamide (**6**) to give gigantamide A (**7**) are outlined in Table 1.

**Table 1:** Synthesis of gigantamide A (**7**) by oxidation of dasyclamide (**6**).

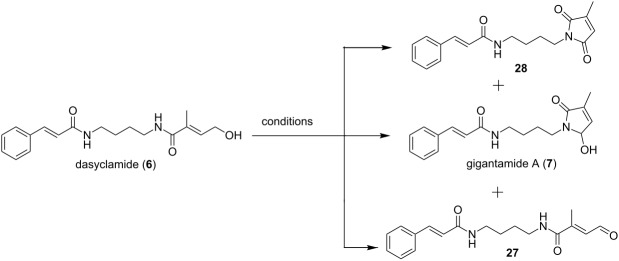		

Entry	Conditions	Results

1	MnO_2_, dioxane, reflux	no reaction
2	TPAP, NMO, ACN/DCM, rt	incomplete reaction & close impurities
3	Py·SO_3_, TEA, DMSO, rt to 80 °C	no reaction
4	PCC, CHCl_3_, rt	incomplete reaction & difficulties in isolation
5	IBX, fluorobenzene/DMSO (9:1), 90 °C, 4 h	70% of aldehyde after purification.
6	PCC, CHCl_3_/DMSO (3:1), rt	30% of aldehyde (**27**) + 25% of gigantamide A + 30% of **28**

The results clearly demonstrated that IBX (Table 1, entry 5) is the best reagent to get the aldehyde **27** in better yield and quality. After analyzing the products obtained from the oxidation with PCC (Table 1, entry 4), we found that the reaction produced gigantamide A (**7**) along with the aldehyde **27**. We have also isolated *N*-(4-(3-methyl-2,5-dioxo-2,5-dihydro-1*H*-pyrrol-1-yl)butyl)cinnamamide (**28**) as one of the products which might have been formed by the oxidation of gigantamide A (**7**) by excess of PCC. Comparison of the NMR data with those reported for the natural gigantamide A (**7**) [7] confirmed the identity of the synthetic product. The overall yield for the synthetic route was found to be 2.3%. Chiral HPLC analysis revealed that it is a racemic compound which was further confirmed by the optical rotation ([α]_D_^23^= 0 (*c*, 0.5, CHCl_3_); reported for the enatio-pure compound: [α]_D_^20^= −10 (*c*, 0.3, CHCl_3_)). Further attempt to get (−)-gigantamide by lipase catalyzed kinetic resolution in line with the reported method for *R*-jatropham [23] was not successful.

## Conclusion

In conclusion, a facile synthesis of (±)-grandiamide D (**5**), dasyclamide (**6**) and gigantamide A (**7**) and asymmetric synthesis of natural (+)-grandiamide D (**5**) was achieved with the aid of a Baylis–Hillman adduct. We have also demonstrated a one-step conversion of dasyclamide (**6**) to gigantamide A (**7**) by oxidative intramolecular cyclization by using PCC.

## Supporting Information

File 1 Detailed experimental procedures, copies of ^1^H and ^13^C NMR spectra for the new compounds and chiral HPLC reports.
