# Supplementary material for: Total synthesis of (+)-grandiamide D, dasyclamide and gigantamide A from a Baylis–Hillman adduct: A unified biomimetic approach
Source: Beilstein J Org Chem. 2014 Jan 10;10:127–33. doi: 10.3762/bjoc.10.9 (PMC3896245; doi:10.3762/bjoc.10.9)
Supplement: File 1 — Detailed experimental procedures, copies of 1H and 13C NMR spectra for the new compounds and chiral HPLC reports. [file Beilstein_J_Org_Chem-10-127-s001.pdf]

Supporting Information

for

Total synthesis of (+)-grandiamide D, dasyclamide and gigantamide A from a Baylis–Hillman adduct: A unified biomimetic approach

Andivelu Ilangovan[1,*], Shanmugasundar Saravanakumar[1,2]

[1]School of Chemistry, Bharathidasan University, Tiruchirappalli, 620024, India and [2]Syngene International Ltd., Bangalore, 560 099, India.

Email: Andivelu Ilangovan[*] - ilangovanbdu@yahoo.com

*Corresponding author

Detailed experimental procedures, copies of ^{1}H and ^{13}C NMR spectra for the new compounds and chiral HPLC reports

Contents

General information	s02
Experimental procedures	s03
References	s16
^{1}H & ^{13}C NMR spectra	s17
Chiral HPLC reports	s40

General information

Nuclear magnetic resonance spectra were recorded on AVANCE-300 MHz and 400 MHz (Bruker) spectrometers using tetramethylsilane (TMS) as the internal standard. Data were reported as follows: chemical shift (multiplicity [singlet (s), doublet (d), doublet of doublet (dd), triplet (t), quartet (q), pentet (p), multiplet (m), broad singlet (brs)], coupling constants [Hz], integration). All the carbon NMR spectra were recorded on (75 & 100 MHz) spectrometers with complete proton decoupling. Carbon chemical shifts were reported in ppm with the respective solvent resonance as the internal standard. Melting points were recorded (uncorrected) on a Büchi Melting Point B-545 instrument. Chiral HPLC was performed with Chiralpak ADH, Chiralpak IC and ChiralCel ODH columns of 250 x 4.6 mm and a Hitachi L-7455 photodiode array detector at 25 °C. The elemental analysis was performed on a Thermo Finnigan EA 1112 CHN analyzer. The mass spectra were recorded on an Agilent LC/MSD SL 1100 instrument. Optical rotations were determined with a Jasco P-200 polarimeter and reported as follows: $[\alpha]_D^{20}$ (*c* g/100 mL, in solvent). Reactions that required inert atmosphere and moisture control were carried out in a nitrogen atmosphere employing oven-dried glassware. All the solvents were used as purchased. All the chemicals were purchased from commercial sources.

Experimental procedures

Synthesis of ethyl 2-((4-methoxybenzyl)oxy)acetate (**13**) [1]

To a dispersion of NaH (60% in mineral oil, 16.00 g, 0.3986 mol) in dry THF (500 mL), 4-methoxybenzylalcohol (50.00 g, 0.3624 mol) was added slowly, at 20–25 °C, over 1 h. The reaction mixture was stirred at room temperature for 1 h. The resulting yellow solution was cooled to 0 °C and ethyl bromoacetate (66.60 g, 0.3624 mol) was added dropwise for 1 h. The mixture was warmed to room temperature and stirred for 20 h. The reaction mixture was quenched with ice water and the product was extracted into EtOAc (3 x 200 mL). The combined EtOAc layer was washed with water (3 x 200 mL), brine (100 mL), dried (anhyd. Na_2SO_4) and evaporated under vacuum to provide the crude product which was purified by flash chromatography over silica (230–400) using EtOAc/hexane (0–20%) as eluent to provide the title compound **13** as colorless oil.

Yield: 48.00 g (59%)

^{1}H NMR (300 MHz, $CDCl_3$): δ 1.31 (t, J = 7.2 Hz, 3H, $OCH_2\underline{CH_3}$), 3.93 (s, 3H, OCH_3), 4.15 (s, 2H, $\underline{CH_2}$Ar), 4.35 (q, J = 7.2 Hz, 2H, $O\underline{CH_2}CH_3$), 6.81 (d, J = 8.7 Hz, 2H, ArH), 7.19 (d, J = 8.7 Hz, 2H, ArH); ^{13}C NMR (75 MHz, $CDCl_3$): δ 14.3, 55.0, 60.4, 66.9, 73.1, 114.0, 129.2, 129.9, 159.8, 171.5.

Spectral data for compound **13** was in agreement with the values reported in the literature [1].

Synthesis of 2-((4-methoxybenzyl)oxy)acetaldehyde (**14**) [2]

To a solution of ethyl 2-((4-methoxybenzyl)oxy)acetate (**13**, 48.00 g, 0.2143 mol) in dry toluene (450 mL) at −78 °C, under N_2 atmosphere, was added DIBAL-H (1.5 M in toluene, 33.60 g, 157 mL, 0.2357 mol) for 1 h, keeping the temperature below −60 °C.

The clear solution was warmed to 0 °C slowly over a period of 1 h after stirring for an additional hour at that temperature. The mixture was quenched by slow addition of MeOH at 0 °C. Water was added and the toluene layer was separated. The organic layer was washed with water (3 x 200 mL), brine (100 mL), dried over anhydrous Na_2SO_4 and evaporated under vacuum to provide the crude aldehyde **14** as pale brown oil which was directly used for the next step.

Yield: 38.00 g (99%)

^1H NMR (300 MHz, $CDCl_3$): *δ* 3.74 (s, 3H, OCH_3), 4.12 (s, 2H, OCH_2), 4.59 (s, 2H, $\underline{CH_2}Ar$), 6.83 (d, *J* = 8.7 Hz, 2H, ArH) 7.23 (d, *J* = 8.7 Hz, 2H, ArH), 10.43 (s, 1H, CHO); ^{13}C NMR (75 MHz, $CDCl_3$): *δ* 55.4, 73.4, 75.1, 114.1, 128.9, 129.8, 159.7, 200.5.

Spectral data for compound **14** was in agreement with the values reported in the literature [2].

Synthesis of (±)-ethyl 3-hydroxy-4-((4-methoxybenzyl)oxy)-2-methylenebutanoate ((±)-16)

A mixture of 2-((4-methoxybenzyl)oxy)acetaldehyde (**14**, 30.00 g, 0.1667 mol), ethyl acrylate (50.00 g, 0.5000 mol) and DABCO (18.60 g, 0.1607 mol) in dioxane (300 mL) and water (300 mL) was stirred at room temperature for 40 h. The reaction mixture was bifurcated between EtOAc (500 mL) and water (1 L). The organic layer was separated, washed with water (3 x 200 mL), brine, dried over anhydrous Na_2SO_4 and evaporated under vacuum. The crude product was purified by flash chromatography over silica (230–400) using EtOAc-hexane (0–15%) as eluent to provide the title compound **(±)-16** as colorless oil.

Yield: 30.00 g (65%)

^{1}H NMR (400 MHz, $CDCl_3$): δ 1.36 (t, J = 7.3 Hz, 3H, $OCH_2\underline{CH_3}$), 3.37 (dd, J = 9.3, 7.1 Hz, 1H, OCH_2), 3.70 (dd, J = 9.6, 7.2 Hz, 1H, OCH_2), 3.80 (s, 3H, OCH_3), 4.21 (q, J = 7.3 Hz, 2H, $O\underline{CH_2}CH_3$), 4.49 (s, 2H, $\underline{CH_2}Ar$), 4.20 (d, J = 8.0 Hz, 1H, OH), 4.71-4.72 (m, 1H, CHOH), 6.01 (d, J = 1.2 Hz, 1H,C =$\underline{CH_2}$), 6.35 (s, 1H, C=$\underline{CH_2}$), 6.89-7-6.91 (m, 2H, ArH), 7.25-7.32 (m, 2H, ArH); ^{13}C NMR (100 MHz, $CDCl_3$): δ 51.8, 55.2, 69.4, 72.9, 73.2, 113.8, 126.6, 128.6, 129.6, 139.0, 159.2, 166.3; Anal. Calcd. for $C_{15}H_{20}O_5$: C, 64.27; H, 7.19, Found: C, 64.23; H, 7.21.

Synthesis of (±)-3-hydroxy-4-((4-methoxybenzyl)oxy)-2-methylenebutanoic acid (±)-(17)

To a solution of (±)-ethyl 3-hydroxy-4-((4-methoxybenzyl)oxy)-2-methylenebutanoate (**(±) 16**, 35.00 g, 0.1250 mol) in THF (400 mL), a solution of LiOH (10.00 g, 0.2450 mol) in water (100 mL) was added slowly for 30 minutes at room temperature. The reaction mixture was stirred for 6 h. THF was removed under vacuum and water was added. The aqueous layer was washed with Et_2O (2 x 100 mL), acidified with 1 N HCl and extracted with EtOAc (3 x 200 mL). The combined EtOAc layer was washed with water (3 x 200 mL), brine, dried over anhydrous Na_2SO_4 and evaporated under vacuum to provide the title compound **(±)-17** as colorless oil.

Yield: 30.00 g (95%)

^{1}H NMR (300 MHz, DMSO-D_6): δ 3.22-3.24 (m, 1H, OCH_2), 3.42-3.43 (m, 1H, OCH_2), 3.72 (s, 3H, OCH_3), 4.39 (s, 2H, $\underline{CH_2}Ar$), 4.50-4.52 (m, 1H, $\underline{CH}OH$), 5.81 (s, 1H, C =$\underline{CH_2}$), 6.11 (s, 1H, C=$\underline{CH_2}$), 6.88 (d, J = 8.4 Hz, 2H, ArH), 7.23 (d, J = 8.4 Hz, 2H, ArH); ^{13}C NMR (100 MHz, $CDCl_3$): δ 55.5, 68.7, 72.2, 74.3, 114.0, 124.7, 129.6, 130.9,

143.1, 159.1, 167.9. LCMS (ESI): m/z 253.2 $[M + H]^+$; Anal. Calcd. for $C_{13}H_{16}O_5$: C, 61.90; H, 6.39, Found: C, 61.93; H, 6.41.

Synthesis of *tert*-Butyl 4-cinnamamidobutylcarbamate (**10**) [3]

To a solution of cinnamic acid (10.00 g, 0.0676 mol) and *tert*-butyl 4-aminobutylcarbamate (12.70 g, 0.0676 mol) in THF (100 mL), a solution of EDCI·HCl (14.30 g, 0.0743 mol) and TEA (7.30 g, 0.0743 mol) in $CHCl_3$ (100 mL) was added slowly for 30 minutes at 0 °C. The mixture was warmed slowly to room temperature and stirred for 19 h. The solvents were removed under reduced pressure and the residue was diluted with EtOAc (500 mL). The combined EtOAc layer was washed with 10% $NaHCO_3$ solution (100 mL), 1 N HCl (100 mL), water (2 x 100 mL), brine, dried over anhydrous Na_2SO_4 and evaporated under vacuum to get the title compound **10** as white solid.

Yield: 18.00 g (82%)

mp: 92.6-96.1 °C; ^{1}H NMR (300 MHz, $CDCl_3$): δ 1.45 (s, 9H, *t*-butyl), 1.45-1.59 (m, 4H, CH_2-CH_2), 3.17-3.44 (m, 4H, CH_2-CH_2),), 4.65 (br s, 1H, NH), 6.13 (br s, 1H, NH), 6.43 (d, J = 15.2 Hz, 1H, PhCH=<u>CH</u>), 7.36-7.38 (m, 3H, ArH), 7.50-7.52 (m, 2H, ArH), 7.64 (d, J = 15.2 Hz, 1H, Ph<u>CH</u>=CH); ^{13}C NMR (100 MHz, $CDCl_3$): δ 26.3, 27.2, 28.7, 39.3, 40.1, 79.3, 120.5, 127.4, 128.6, 129.9, 135.2, 140.8, 156.6, 166.5.

Spectral data for compound **10** was in agreement with the values reported in the literature [3].

Synthesis of *N*-(4-aminobutyl)cinnamamide (**11**) [3]

A solution of *tert*-butyl 4-cinnamamidobutylcarbamate (**10**, 18.00 g) in EtOH (180 mL) was treated with 6 N HCl (90 mL) and the mixture was stirred at 50 °C for 3 h. The EtOH

was distilled off and the residue was basified 2 N NaOH solution to pH 14 and extracted with DCM (4 x 100 mL). The combined organic layer was dried over anhydrous Na_2SO_4 and concentrated under reduced pressure to provide the title compound **11** as pale brown solid.

Yield: 11.00 g (89%)

mp: 79.3-81.7 °C; ^{1}H NMR (300 MHz, $CDCl_3$): δ 1.45 (s, 9H, *t*-butyl), 1.41-1.65 (m, 4H, CH_2-CH_2), 2.85-3.45 (m, 4H, CH_2-CH_2), 6.31 (br s, 1H, NH), 6.41 (d, *J* = 15.2 Hz, 1H, PhCH=<u>CH</u>), 7.31 (m, 3H, ArH), 7.49 (m, 2H, ArH), 7.66 (d, *J* = 15.4 Hz, 1H, Ph<u>CH</u>=CH); ^{13}C NMR (100 MHz, $CDCl_3$): δ 26.3, 27.2, 28.7, 39.3, 40.1, 79.3, 120.5, 127.4, 128.6, 129.9, 135.2, 140.8, 156.6, 166.5.

Spectral data for compound **11** was in agreement with the values reported in the literature [3].

Synthesis of (±)-(*E*)-*N*-(4-cinnamamidobutyl)-3-hydroxy-4-((4-methoxybenzyl)oxy)-2 methylenebutanamide (±)-(18)

To a solution of (±)-3-hydroxy-4-(4-methoxybenzyloxy)-2-methylenebutanoic acid (**(±)-17**, 5.00 g, 0.0199 mol) *N*-(4-aminobutyl)cinnamamide (**11**, 4.35 g, 0.0199 mol) in THF (50 mL), a solution of EDCI·HCl (4.58 g, 0.0238 mol) and TEA (4.82 g, 0.0288 mol) in $CHCl_3$ (50 mL) was added slowly for 30 minutes at 0 °C. The mixture was warmed slowly to room temperature and stirred for 19 h. The solvents were removed under reduced pressure and the residue was extracted with EtOAc (3 x 50 mL). The combined EtOAc layer was washed with 10% $NaHCO_3$ solution (50 mL), 1 N HCl (50 mL), water (2 x 50 mL), brine, dried over anhydrous Na_2SO_4 and evaporated in vacuum. The crude

product was crystallized with DCM/Et_2O to get the title compound **(±)-18** as an off-white solid.

Yield: 6.00 g (67%)

mp: 238.4-241.5 °C; ^{1}H NMR, (400 MHz, DMSO-d_6): δ 1.44-1.48 (m, 4H, CH_2-CH_2), 3.10-3.28 (m, 1H, O$\underline{CH_2}$), 3.39-3.42 (m, 1H, O$\underline{CH_2}$), 3.71 (s, 3H, OCH_3), 4.38 (s, 2H, CH_2Ar), 4.57-4.52 (m, 1H, $\underline{CH}$OH), 5.22-5.23 (d, J = 4.0 Hz, 1H, CH$\underline{OH}$), 5.53 (s, 1H, C=$\underline{CH_2}$), 5.69 (s, 1H, C=$\underline{CH_2}$) 6.61 (d, J = 16.0 Hz, 1H, PhCH=$\underline{CH}$), 6.86 (d, J = 8.5 Hz, 2H, ArH), 7.22 (d, J = 8.5 Hz, 2H, ArH), 7.35 (d, J = 16.0 Hz, 1H, Ph$\underline{CH}$=CH), 7.36-7.37 (m, 3H, ArH), 7.53-7.54 (m, 2H, ArH), 8.02 (t, J = 4.0 Hz, 1H, NH), 8.10 (t, J = 4.0 Hz, 1H, NH); ^{13}C NMR (100 MHz, DMSO-d_6): δ 27.1, 27.1, 38.7, 38.9, 55.5, 69.3, 72.2, 74.1, 113.9, 117.9, 122.8, 127.9, 129.4, 129.6, 130.8, 135.4, 138.9, 146.23, 159.1, 165.3, 167.5; Anal. Calcd. for $C_{26}H_{32}N_2O_5$: C, 69.01; H, 7.13; N, 6.19, Found: C, 69.05; 7.09; N, 6.21.

Synthesis of (±)-grandiamide D (**5**) [4]

To a solution of (±)-(*E*)-*N*-(4-cinnamamidobutyl)-3-hydroxy-4-((4-methoxybenzyl)oxy)-2 methylenebutanamide (**(±)-18**, 2.00 g) in DCM (50 mL), TFA (2 mL) was added slowly for 10 minutes at room temperature. The reaction mixture was stirred for 5 h. The reaction mixture was quenched with 10% NaOH solution (10 mL), the DCM layer was separated, washed with water (2 x 50 mL), brine and dried over anhydrous Na_2SO_4. The solvent was removed under reduced pressure and the crude product was purified by column chromatography over silica gel (230–400) using $CHCl_3$/MeOH (0–5%) as eluent to get (±)-grandiamide D (**5**) as white solid.

Yield: 1.10 g (75%)

^{1}H NMR (400 MHz, CD_3OD): δ 1.58-1.60 (m, 4H, CH_2-CH_2), 3.27-3.32 (m, 4H, CH_2-CH_2), 3.48 (dd, J = 11.1, 6.9 Hz, 1H, $\underline{CH_2}$OH), 3.63 (dd, J = 11.1, 6.9 Hz, 1H, $\underline{CH_2}$OH), 4.51 (t, J = 5.5 Hz, 1H, $\underline{CH}$OH), 5.61 (s, 1H, C=$\underline{CH_2}$), 5.78 (s, 1H, C=$\underline{CH_2}$), 6.59 (d, J = 15.8 Hz, 1H, PhCH=$\underline{CH}$), 7.34-7.37 (m, 3H, ArH), 7.39 (d, J = 15.8 Hz, 1H, Ph$\underline{CH}$=CH), 7.52-7.55 (m, 2H, ArH); ^{13}C NMR (100 MHz, CD_3OD): δ: 27.7, 27.8, 39.9, 40.1, 66.6, 73.2, 119.8, 121.8, 128.7, 129.8, 130.7, 136.2, 141.5, 146.3, 168.5, 170.4; LCMS (ESI): m/z 333.3 $[M + H]^+$.

Spectral data for compound **5** was in agreement with the values reported in the literature [4].

Synthesis of (*S*)-ethyl 3-hydroxy-4-((4-methoxybenzyl)oxy)-2-methylenebutanoate (+)-16

To a solution of acryloyl sultam **19** (478 mg, 1.780 mmol) and DABCO (19.80 mg, 0.1800 mmol) in DMF (5 mL), at 0 °C, was added 2-(4-methoxybenzyloxy)acetaldehyde (**14**, 4.67 g, 25.9 mmol) and the solution was stirred at room temperature for 120 h. The reaction mixture was diluted with EtOAc (100 mL), washed with water (3 x 100 mL) brine and dried over anhydrous Na_2SO_4. The solvent was removed under reduced pressure and the crude product was dissolved in ethanol (100 mL) containing TEA (5 mL). The mixture was stirred at room temperature for 6 h, evaporated the solvent under reduced pressure and the crude product was purified by purified by flash chromatography over silica (230-400) using EtOAc-hexane (0–15%) as eluent to provide the title compound **(+)-16** as colorless oil.

Yield: 353 mg (71%)

$[\alpha]_D^{25}$ = +14.1 (*c* 1.0, $CHCl_3$); Chiralpak AD-H, 0.1% TFA in hexane/ethanol (80:20), 1.0 mL\min, λ = 206 nm, *t*R = 8.2 minor, *t*R = 10.6 major

Synthesis of (*S*)-3-hydroxy-4-(4-methoxybenzyl)oxy)-2-methylenebutanoic acid (+)-17

The ester **(+)-16** was hydrolyzed with LiOH as described earlier in the synthesis of compound **(±)-17** to afford the title compound **(+)-17** as pale yellow oil.

$[\alpha]_D^{25}$ = +16.97 (*c* 0.5, $CHCl_3$); Chiralpak AD-H, 0.1% TFA in hexane/ethanol (80:20), 1.0 mL\min, λ = 206 nm, *t*R = 10.7 minor, *t*R = 14.5 major.

Synthesis of (*S,E*)-*N*-(4-cinnamamidobutyl)-3-hydroxy-4-((4-methoxybenzyl)oxy)-2-methylenebutanamide (+)-18

The acid **(+)-17** was coupled with *N*-(4-aminobutyl)cinnamamide (**11**) as decribed earlier in the synthesis of compound **(±)-18** to obtain the title compound **(+)-18**.

$[\alpha]_D^{25}$ = +10.45 (*c* 0.5, $CHCl_3$); Chiralpak IC, hexane/ethanol (70:30), 1.0 mL\min, λ = 206 nm, *t*R = 10.7 minor, *t*R = 14.5 major.

Synthesis of (+)-grandiamide D (**5**) **[4]**

Compound **(+)-18** was deprotected with TFA as described earlier in the synthesis of (±)-grandiamide D (**5**) to afford (+)-grandiamide D (**5**).

$[\alpha]_D^{26}$ = +4.76 (*c* 0.5, MeOH); Chiralpak IC, hexane/ethanol (70:30), 1.0 mL\min, λ = 206 nm, *t*R = 5.8 minor, *t*R = 7.1 major.

Synthesis of (±) ethyl 3-acetoxy-4-(4-methoxybenzyloxy)-2-methylenebutanoate (±)-23

To a solution of (±)-ethyl 3-hydroxy-4-((4-methoxybenzyl)oxy)-2-methylenebutanoate (13.00 g, 0.0466 mol) in dry DCM (150 mL), pyridine (7.36 g, 0.0932 mol) was added

and the clear solution was cooled to 0 °C. Acetyl chloride (7.36 g, 0.0932 mol) was added slowly for 20 minutes. The reaction mixture was warmed to room temperature slowly over a period of 30 minutes. The reaction mixture was washed with 1.5 N HCl (50 mL), water (2 x 50 mL), brine (50 mL), dried over anhydrous Na_2SO_4 and evaporated under vacuum to get the desired product (±)-**23** as pale yellow oil.

Yield: 14.60 g (95%)

^{1}H NMR (300 MHz, $CDCl_3$): δ 1.28 (t, *J* = 7.2 Hz, 3H, $OCH_2\underline{CH_3}$), 2.12 (s, 3H, $OC(O)\underline{CH_3}$), 3.58-3.69 (m, 2H, OCH_2), 3.81 (s, 3H, OCH_3), 4.21 (q, *J* = 7.2 Hz, 2H, $O\underline{CH_2}CH_3$), 4.50 (q, *J* = 9.0 Hz, 2H, $\underline{CH_2}Ar$), 5.85 (m, 1H, OCH), 5.88 (t, *J* = 3.0 Hz, 1H, $C{=}\underline{CH_2}$), 6.36 (s, 1H, $C{=}\underline{CH_2}$), 6.86-6.90 (m, 2H, ArH), 7.23-7.27 (m, 2H, ArH); ^{13}C NMR (75 MHz, $CDCl_3$): δ 14.0, 21.0, 55.2, 60.9, 70.4, 70.7, 70.5, 113.7, 126.6, 129.2, 129.9, 137.0, 159.2, 164.9, 169.7; Anal. Calcd. for $C_{17}H_{22}O_6$: C, 63.34; H, 6.88, Found: C, 63.38; H, 6.85.

Synthesis of (E)-ethyl 4-(4-methoxybenzyloxy)-2-methylbut-2-enoate (**24**) [2]

$NaBH_4$ (3.26 g, 0.0860 mol) was added in portions to a solution of (±)-ethyl 3-acetoxy-4-((4-methoxybenzyl)oxy)-2-methylenebutanoate (**23**, 14.50 g, 0.0430 mol) in dry *tert*-butanol (150 mL) over a period of 30 minutes at room temperature. The reaction mixture was stirred for 19 h. The reaction mixture was diluted with water and the product was extracted with EtOAc (2 x 150 mL). The combined EtOAc layer was washed with water (3 x 200 mL), brine, dried over anhydrous Na_2SO_4 and evaporated under vacuum to provide the crude product **24** as colorless oil which was pure enough to take into the next step.

Yield: 10.50 g (91%)

^{1}H NMR (300 MHz, $CDCl_3$): δ 1.26 (t, J = 7.2 Hz, 3H, $OCH_2\underline{CH_3}$), 1.82 (s, 3H, $C(\underline{CH_3})CO_2Et$), 3.80 (s, 3H, OCH_3), 4.17 (m, 4H, OCH_2, $O\underline{CH_2}CH_3$), 4.48 (s, 2H, $\underline{CH_2}Ar$), 6.85-6.92 (m, 3H, olefinic, ArH), 7.28 (d, J = 8.7 Hz, 2H, ArH); ^{13}C NMR (75 MHz, $CDCl_3$): δ 12.8, 14.2, 55.2, 60.6, 66.4, 72.4, 113.8, 129.2, 129.3, 129.8, 137.9, 159.3, 167.5.

Spectral data for compound **24** was in agreement with the values reported in the literature [2].

Synthesis of (*E*)-4-(4-methoxybenzyloxy)-2-methylbut-2-enoic acid (**25**)

To a solution of (*E*)-ethyl 4-((4-methoxybenzyl)oxy)-2-methylbut-2-enoate (**24**, 7.60 g, 0.0289 mol) in MeOH (100 mL), a solution of KOH (3.24 g, 0.0570 mol) in water (40 mL) was added slowly for 30 minutes at room temperature. The reaction mixture was stirred for 3 h. Methanol was removed under vacuum and the residue was diluted with water. The aqueous layer was washed with Et_2O (2 x 50 mL), acidified with 1 N HCl and extracted with EtOAc (3 x 50 mL). The combined EtOAc layer was washed with water (3 x 50 mL), brine, dried over Na_2SO_4 and evaporated under vacuum to provide the crude product as colorless oil **25** which was directly taken for the next step.

Yield: 5.80 g (85%)

^{1}H NMR (400 MHz, DMSO-d_6): δ 1.74 (s, 3H, $C(\underline{CH_3})CO_2H$), 3.74 (s, 3H, OCH_3), 4.14 (d, J = 8.0 Hz, 2H, OCH_2), 4.41 (s, 2H, $\underline{CH_2}Ar$), 6.67 (t, J = 4.0 Hz, 1H, olefinic), 6.89-6.92 (m, 2H, ArH), 7.23-7.27 (m, 2H, ArH), 12.37 (s, 1H, COOH); ^{13}C NMR (100 MHz, DMSO-d_6): δ 13.1, 55.5, 66.5, 71.9, 114.1, 129.6, 129.7, 130.4, 138.1, 157.2, 168.8; LCMS (ESI): m/z 234.8 $[M-H]^+$; Anal. Calcd. for $C_{17}H_{22}O_6$: C, 66.09; H, 6.83, Found: C, 66.13; H, 6.88.

Synthesis of (*E*)-*N*-(4-cinnamamidobutyl)-4-((4-methoxybenzyl)oxy)-2-methylbut-2-enamide (26)

To a solution of (*E*)-4-((4-methoxybenzyl)oxy)-2-methylbut-2-enoic acid (**25**, 4.00 g, 0.0170 mol) *N*-(4-aminobutyl)cinnamamide (3.72 g, 0.0170 mol) in THF (50 mL), a solution of EDCI·HCl (3.92 g, 0.0204 mol) and TEA (2.58 g, 0.0255 mol) in $CHCl_3$ (50 mL) was added slowly for 30 minutes at 0 °C. The mixture was warmed to room temperature and stirred for 19 h. The solvents were removed under reduced pressure and the residue was extracted with EtOAc (3 x 50 mL). The combined EtOAc layer was washed with 10% $NaHCO_3$ solution (50 mL), 1 N HCl (50 mL), water (2 x 50 mL), brine, dried over anhydrous Na_2SO_4 and evaporated under vacuum. The crude product was crystallized with Et_2O to get the title compound **26** as pale brown solid.

Yield: 5.00 g (70%)

mp: 228.2-237.5 °C; ^{1}H NMR (400 MHz, DMSO-d_6): δ 1.43-1.44 (m, 4H, CH_2-CH_2), 1.71 (s, 3H, C($\underline{CH_3}$)CO), 3.08-3.16 (m, 4H, CH_2-CH_2), 3.72 (s, 3H, OCH_3), 4.09 (d, *J* = 5.9 Hz, 2H, OCH_2), 4.39 (s, 2H, $\underline{CH_2}$Ar), 6.27 (t, *J* = 4.0 Hz, 1H, olefinic), 6.60 (d, *J* = 16.0 Hz, 1H, PhCH=$\underline{CH}$), 6.88 (dd, *J* = 8.0, 12.0 Hz, 2H, ArH), 7.24 (d, *J* = 8.2 Hz, 2H, ArH), 7.36 (d, *J* = 16.0 Hz, 1H, Ph$\underline{CH}$=CH), 7.37-7.41 (m, 3H, ArH), 7.53-7.55 (m, 2H, ArH), 7.91 (t, *J* = 4.0 Hz, 1H, NH), 8.10 (t, *J* = 4.0 Hz, 1H, NH); ^{13}C NMR (100 MHz, DMSO-d_6): δ 13.5, 27.1, 38.9, 55.5, 66.4, 71.7, 114.1, 122.8, 127.9, 129.4, 129.77, 129.81, 130.6, 131.1, 133.4, 135.4, 138.8, 159.2, 165.2, 168.3; Anal. Calcd. for $C_{26}H_{32}N_2O_4$: C, 71.53; H, 7.39; N, 6.42; Found: C, 71.58; H, 7.41; N, 6.49.

Synthesis of dasyclamide (6) [4]

To a solution of (*E*)-*N*-(4-cinnamamidobutyl)-4-((4-methoxybenzyl)oxy)-2-methylbut-2-enamide (**26**, 4.00 g) in DCM (75 mL), TFA (4 mL) was added slowly for 10 minutes at room temperature. The mixture was stirred at room temperature for 4 h. 10% NaOH solution (20 mL) was added, DCM layer was separated, washed with water (2 x 50 mL), brine, dried over anhydrous Na_2SO_4 and evaporated in vacuum to get the crude product which was crystallized with Et_2O to get the title compound as an off-white solid.

Yield: 1.80 g (60%)

^{1}H NMR (400 MHz, CD_3OD): δ 1.59-1.62 (m, 4H, CH_2-CH_2), 1.83 (s, 3H, C($\underline{CH_3}$)CO), 3.28-3.31 (m, 4H, CH_2-CH_2), 4.24 (d, *J* = 4.0 Hz, 2H, $\underline{CH_2}$OH), 6.34 (t, *J* = 1.2 Hz, 1H, olefinic), 6.62 (d, *J* = 16 Hz, PhCH=$\underline{CH}$), 7.36-7.41 (m, 3H, ArH), 7.51-7.57 (m, 3H, ArH, Ph$\underline{CH}$=CH); ^{13}C NMR (100 MHz, CD_3OD): δ 13.0, 27.9, 40.2, 59.5, 121.9, 128.8, 129.9, 130.8, 133.0, 135.8, 136.3, 141.6, 168.6, 172.0; LCMS (ESI): m/z 317.2 $[M+H]^+$.

Spectral data for compound **6** was in agreement with the values reported in the literature [4].

Synthesis of gigantamide A (**7**) **[4]**

To a solution of dasyclamide (**6**, 100 mg, 0.0003345 mol) in a mixture of DMSO (1 mL) and $CHCl_3$ (2 mL), at 0 °C, was added pyridinium chlorochromate (180 mg, 0.000836 mol) in one lot. The orange solution was warmed to room temperature and stirred for 20 h. The reaction mixture was poured into a mixture of 1 N HCl (5 mL) and $CHCl_3$ (10 mL). The organic layer was separated, washed with water (2 x 25 mL), brine (10 mL), dried over Na_2SO_4 and concentrated under reduced pressure to brown solid. Purification by preparative TLC, using $CHCl_3$/MeOH (9/1) as solvent system, afforded three

compounds. Top spot, appeared in the TLC was *N*-(4-(3-methyl-2,5-dioxo-2,5-dihydro-1*H*-pyrrol-1-yl)butyl)cinnamamide (**28**) obtained as white solid. Next spot, gigantamide A (**7**) was obtained (25 mg, 25%) as an off-white solid. Next lower spot was compound (*E*)-*N*-(4-cinnamamidobutyl)-2-methyl-4-oxobut-2-enamide (**27**), obtained as white solid. mp: 139.6-142.4 °C; ^{1}H NMR (400 MHz, CD_3OD): δ 1.56-1.71 (m, 4H, CH_2-CH_2), 1.86 (s, 3H, C($\underline{CH_3}$)CO), 3.31-3.56 (m, 2H, CH_2), 3.35-3.36 (m, 2H, CH_2), 5.35 (d, *J* = 8.8 Hz, 1H, $\underline{CH}$OH), 6.60 (d, *J* = 16 Hz, 1H, PhCH=$\underline{CH}$), 6.64 (dd, *J* = 1.6, 3.3 Hz, 1H, olefinic), 7.37-7.39 (m, 3H, ArH), 7.50 (d, *J* = 16 Hz, 1H, Ph$\underline{CH}$=CH), 7.54-7.57 (m, 2H, ArH); ^{13}C NMR (100 MHz, DMSO-d_6): δ 10.8, 25.6, 26.8, 38.9, 39.3, 82.1, 120.5, 127.8, 128.8, 129.7, 134.7, 136.6, 138.4, 141.1, 166.7, 170.7; LCMS (ESI): m/z 317.3 $[M+H]^+$.

Spectral data for compound **7** was in agreement with the values reported in the literature [4].

(*E*)-*N*-(4-Cinnamamidobutyl)-2-methyl-4-oxobut-2-enamide (27)

mp: 101.4-104.4 °C; ^{1}H NMR (300 MHz, DMSO-D_6): δ 1.47-1.48 (m, 4H, CH_2-CH_2), 2.26 (s, 3H, C($\underline{CH_3}$)CO), 3.13-3.18 (m, 4H, CH_2-CH_2), 6.28 (dd, *J* = 1.4, 7.7 Hz, 1H, $\underline{CH}$-CHO), 6.62 (d, *J* = 16 Hz, 1H, PhCH=$\underline{CH}$), 7.34-7.43 (m, 4H, ArH, Ph$\underline{CH}$=CH), 7.54-7.56 (m, 2H, ArH), 8.11 (t, *J* = 5.3 Hz, 1H, NH), 8.38 (t, *J* = 5.3 Hz, 1H, NH), 10.11 (d, *J* = 7.5 Hz, 1H, CHO); ^{13}C NMR (75 MHz, CD_3OD): δ 10.8, 27.0, 40.1, 40.5, 121.9, 128.9, 130.5, 130.8, 140.9, 141.6, 152.7, 168.6, 172.4, 193.6; LCMS (ESI): m/z 317.3 $[M+H]^+$.

***N*-(4-(3-Methyl-2,5-dioxo-2,5-dihydro-1*H*-pyrrol-1-yl)butyl)cinnamamide (28)**

mp: 112.6-116.1 °C; ^{1}H NMR (300 MHz, $CDCl_3$): δ 1.55-1.62 (m, 4H, CH_2-CH_2), 2.09 (s, 3H, C($\underline{CH_3}$)CO), 3.39-3.36 (m, 4H, CH_2-CH_2), 5.80 (s, 1H,NH), 6.32 (d, *J* = 5.5 Hz, 1H, olefinic), 6.39 (d, *J* = 15.6 Hz, 1H, PhCH=$\underline{CH}$), 7.35-7.41 (m, 3H, ArH), 7.49-7.52

(m, 2H, ArH), 7.62 (d, *J* = 15.6 Hz, 1H, PhCH=CH); ^{13}C NMR (100 MHz, DMSO-d_6): δ 10.9, 26.1, 26.6, 39.1, 120.6, 127.2, 127.7, 128.7, 129.5, 134.8, 140.8, 145.6, 165.9, 170.9, 171.9; LCMS (ESI): m/z 315.3 $[M+H]^+$; Anal. Calcd. for $C_{18}H_{20}N_2O_3$: C, 69.21; H, 6.45; N, 8.97, Found: C, 69.28; H, 6.41; N, 8.89

References

1. Shintou, T.; Mukaiyama, T. *J. Am. Chem. Soc*. **2004**, *126*, 7359-7367.

2. Ghilagaber, S.; Hunter, W. N.; Marquez, R. *Org. Biomol. Chem*. **2007**, *5*, 97-102.

3. Hesse, M.; Detterbeck, R. *Tetrahedron* **2002**, *58*, 6887-6893.

4. Duong, T. N.; Edrada, R. A.; Ebel, R.; Wray, V.; Frank, W.; Duong, A. T.; Lin, W. H.; Proksch, P. *J. Nat. Prod*. **2007**, *70*, 1640-1643.

^{1}H & ^{13}C NMR Spectra and other data

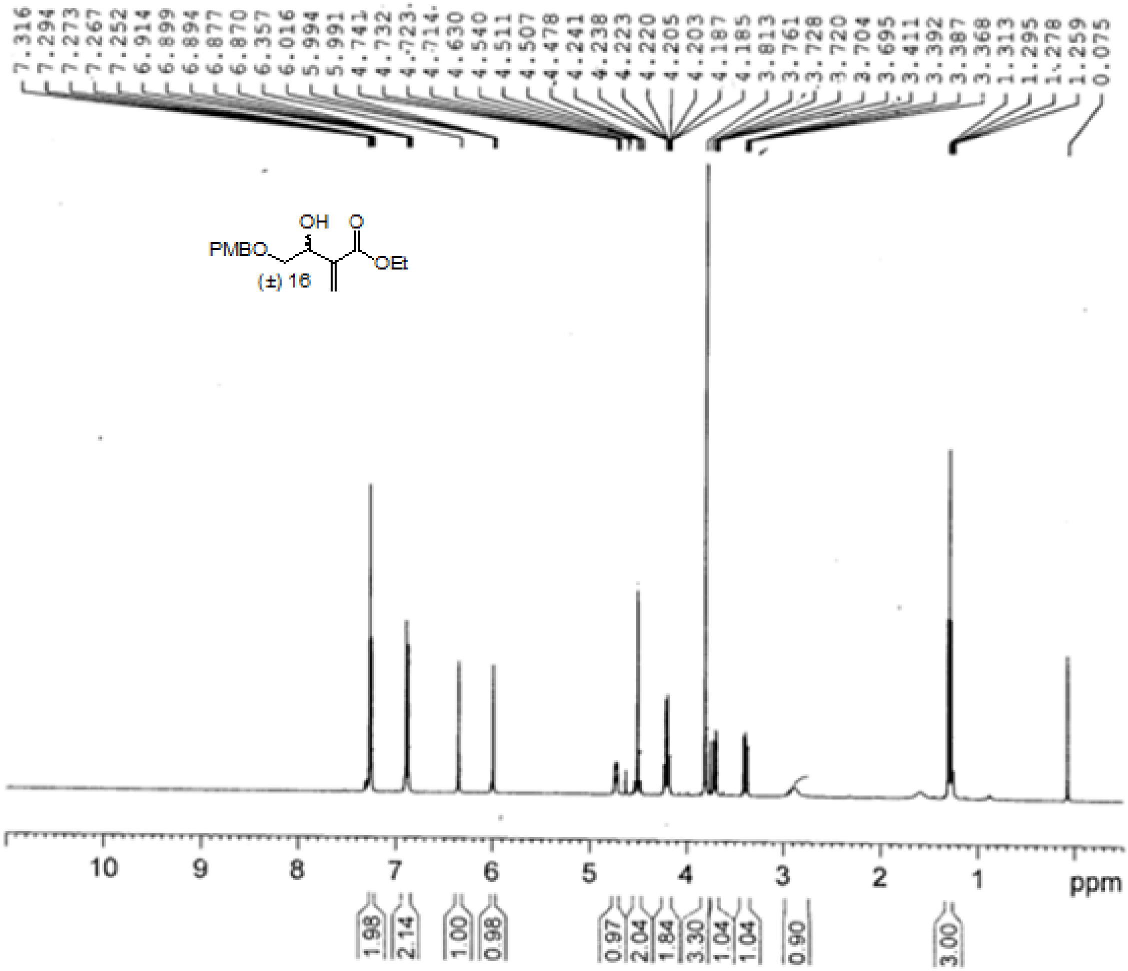

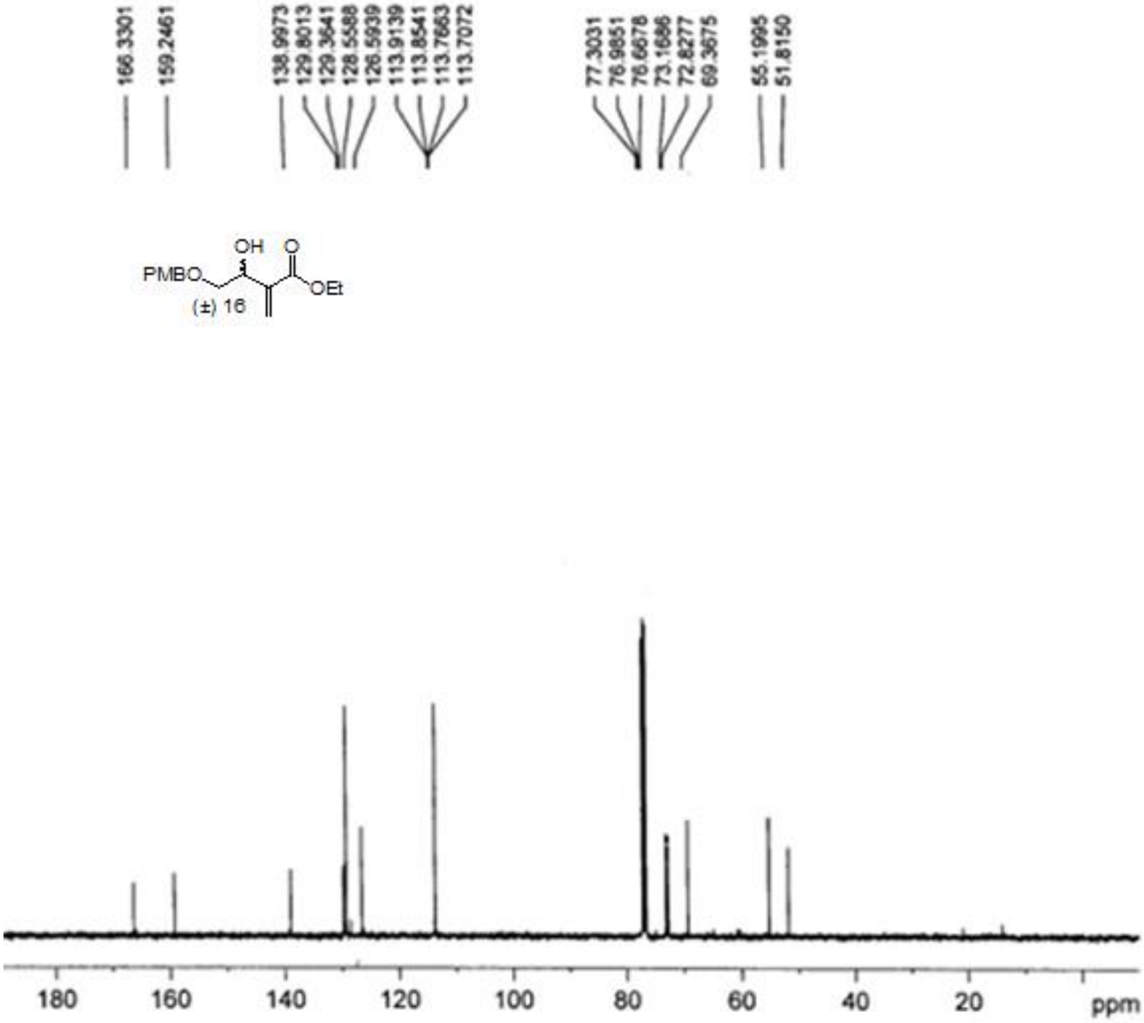
166.3301
159.2461
138.9973
129.8013
129.3641
128.5588
126.5939
113.9139
113.8541
113.7663
113.7072
77.3031
76.9851
76.6678
73.1686
72.8277
69.3675
55.1995
51.8150
PMBO
OH
O
OEt
(±) 16
180
160
140
120
100
80
60
40
20
ppm

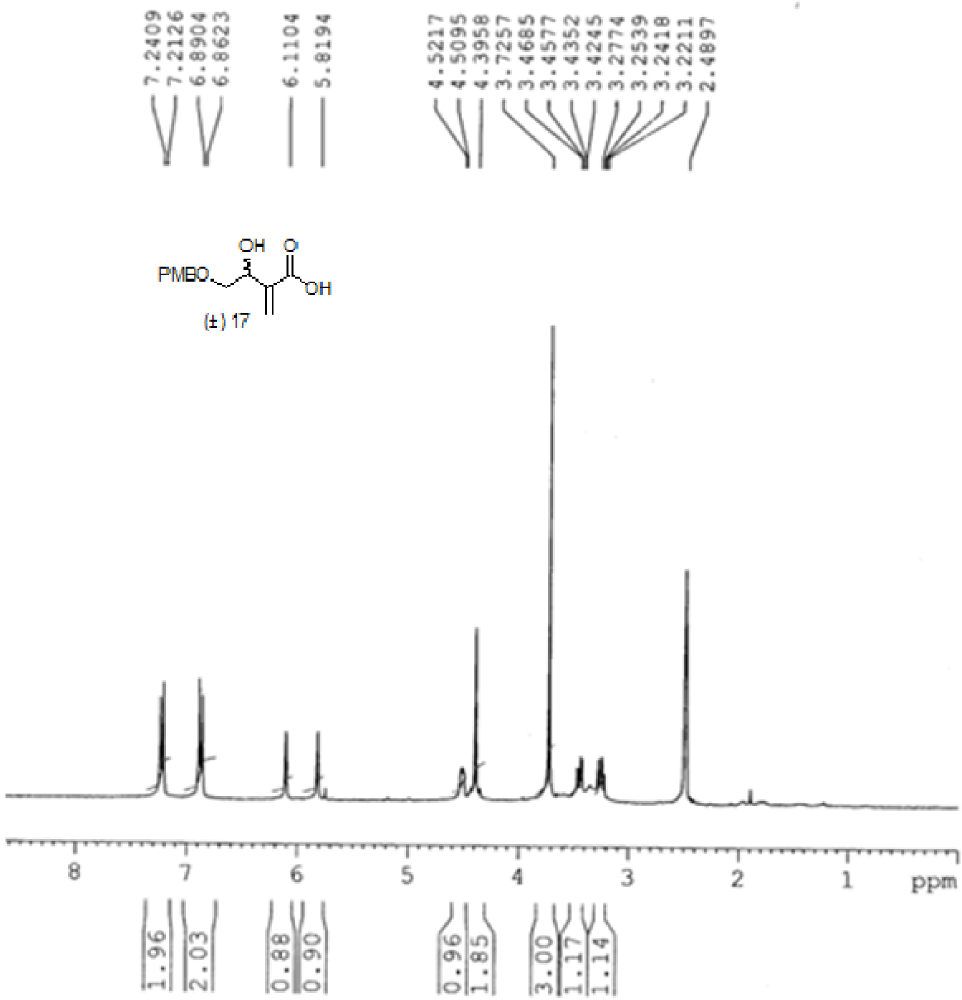
7.2409
7.2126
6.8904
6.8623
6.1104
5.8194
4.5217
4.5095
4.3958
3.7257
3.4685
3.4577
3.4352
3.4245
3.2774
3.2539
3.2418
3.2211
2.4897
OH
O
PMBO
OH
(±) 17
8
7
6
5
4
3
2
1
ppm
1.96
2.03
0.88
0.90
0.96
1.85
3.00
1.17
1.14

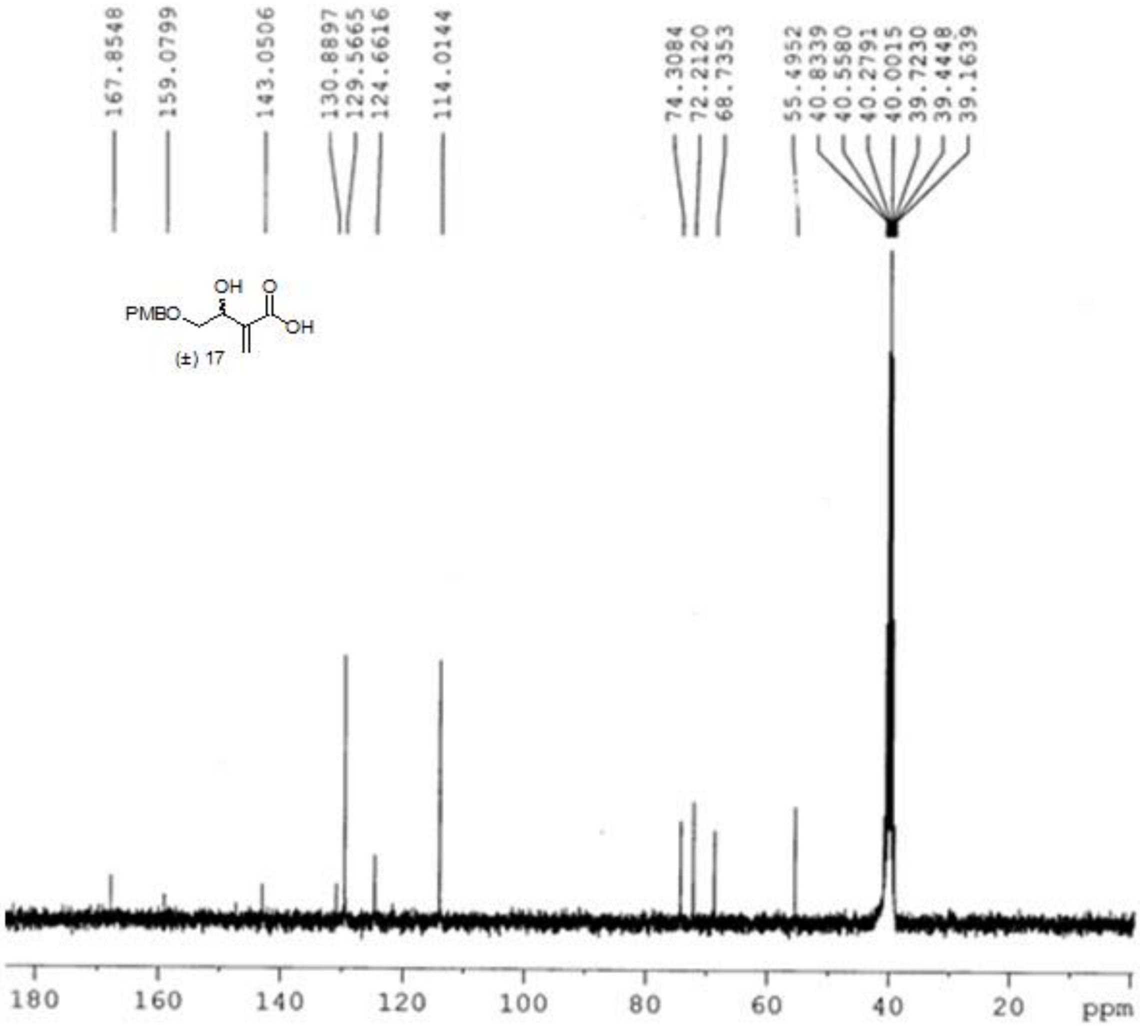
167.8548
159.0799
143.0506
130.8897
129.5665
124.6616
114.0144
74.3084
72.2120
68.7353
55.4952
40.8339
40.5580
40.2791
40.0015
39.7230
39.4448
39.1639
PMBO
OH
O
OH
(±) 17
180
160
140
120
100
80
60
40
20
ppm

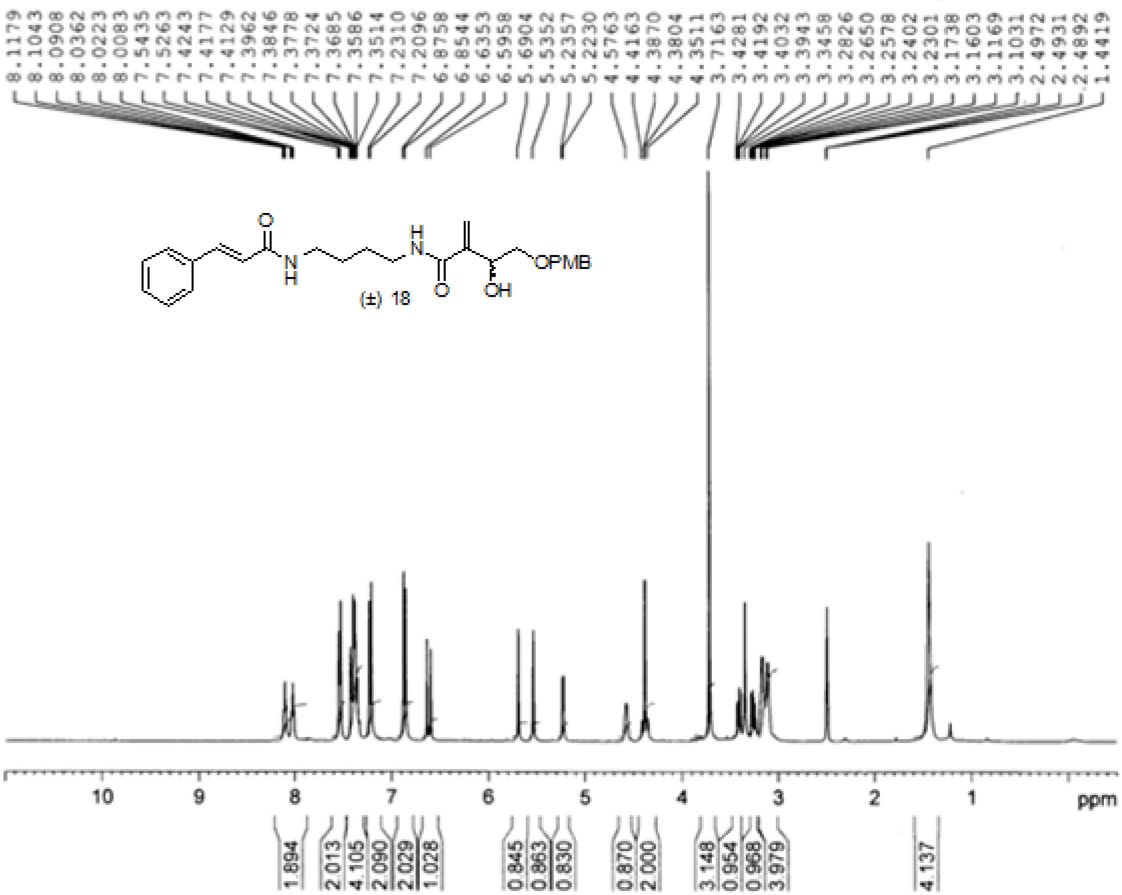

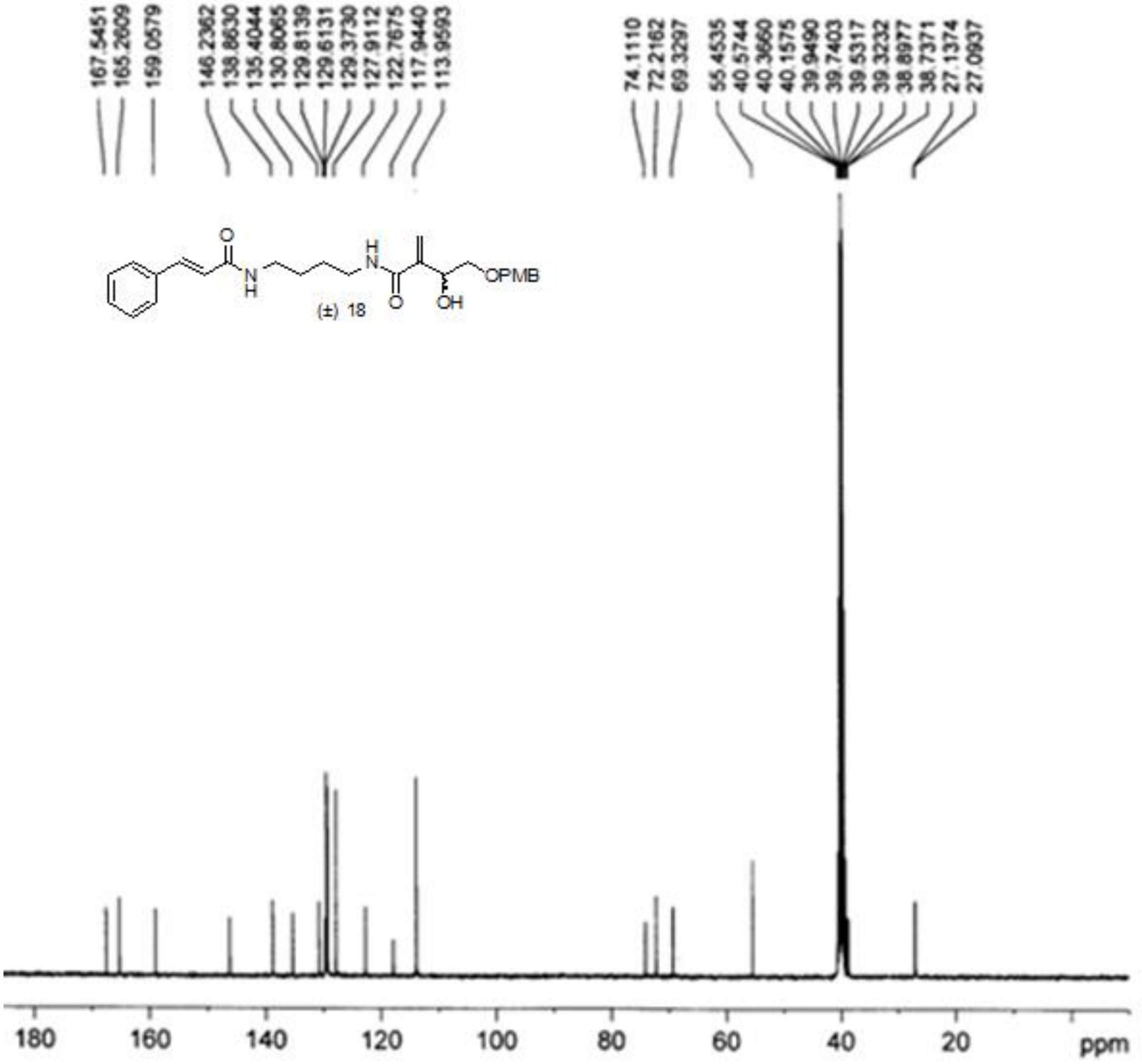
167.5451
165.2609
159.0579
146.2362
138.8630
135.4044
130.8065
129.8139
129.6131
129.3730
127.9112
122.7675
117.9440
113.9593
74.1110
72.2182
69.3297
55.4535
40.5744
40.3660
40.1575
39.9490
39.7403
39.5317
39.3232
38.8977
38.7371
27.1374
27.0937
OPMB
OH
(±) 18
180
160
140
120
100
80
60
40
20
ppm

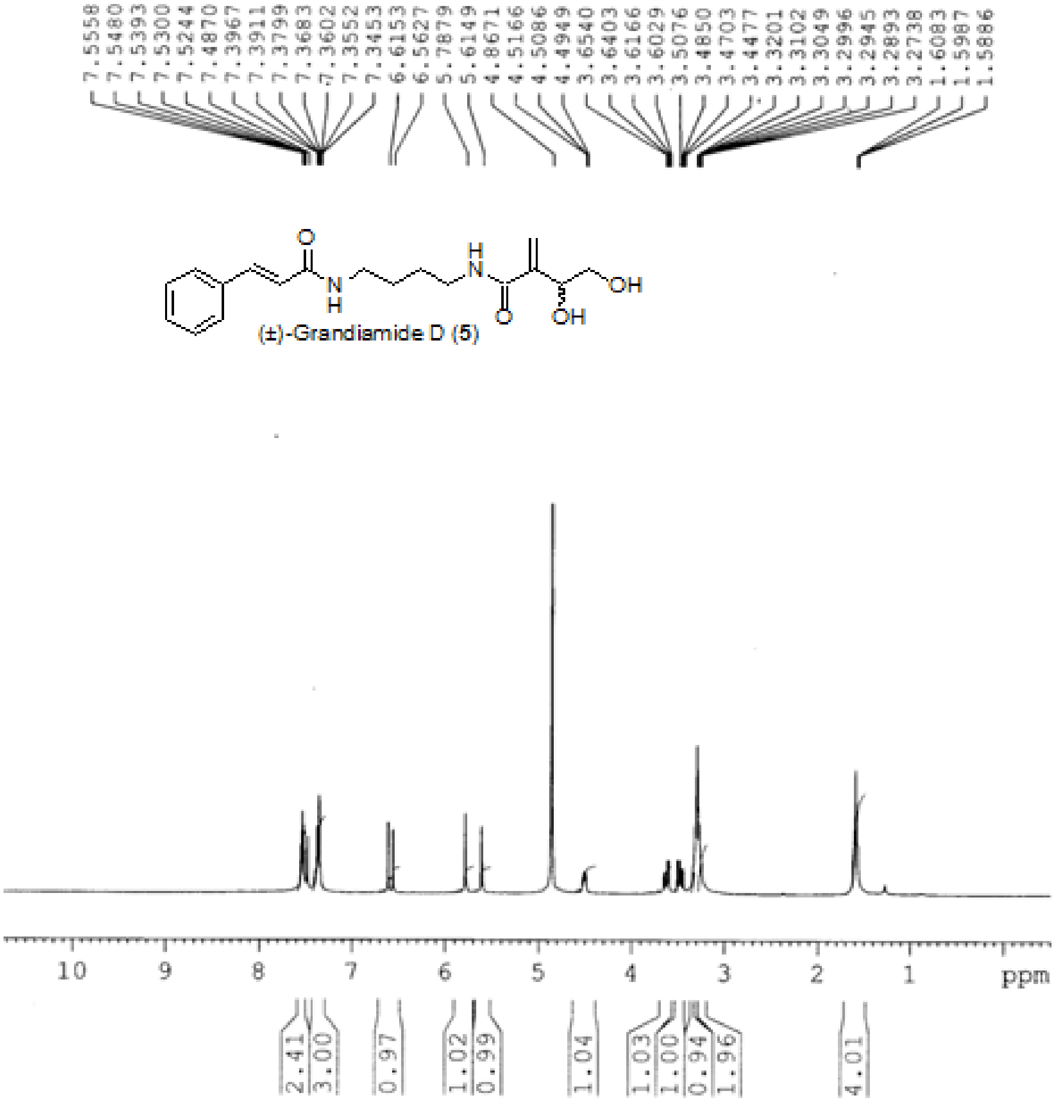
(±)-Grandiamide D (5)
ppm

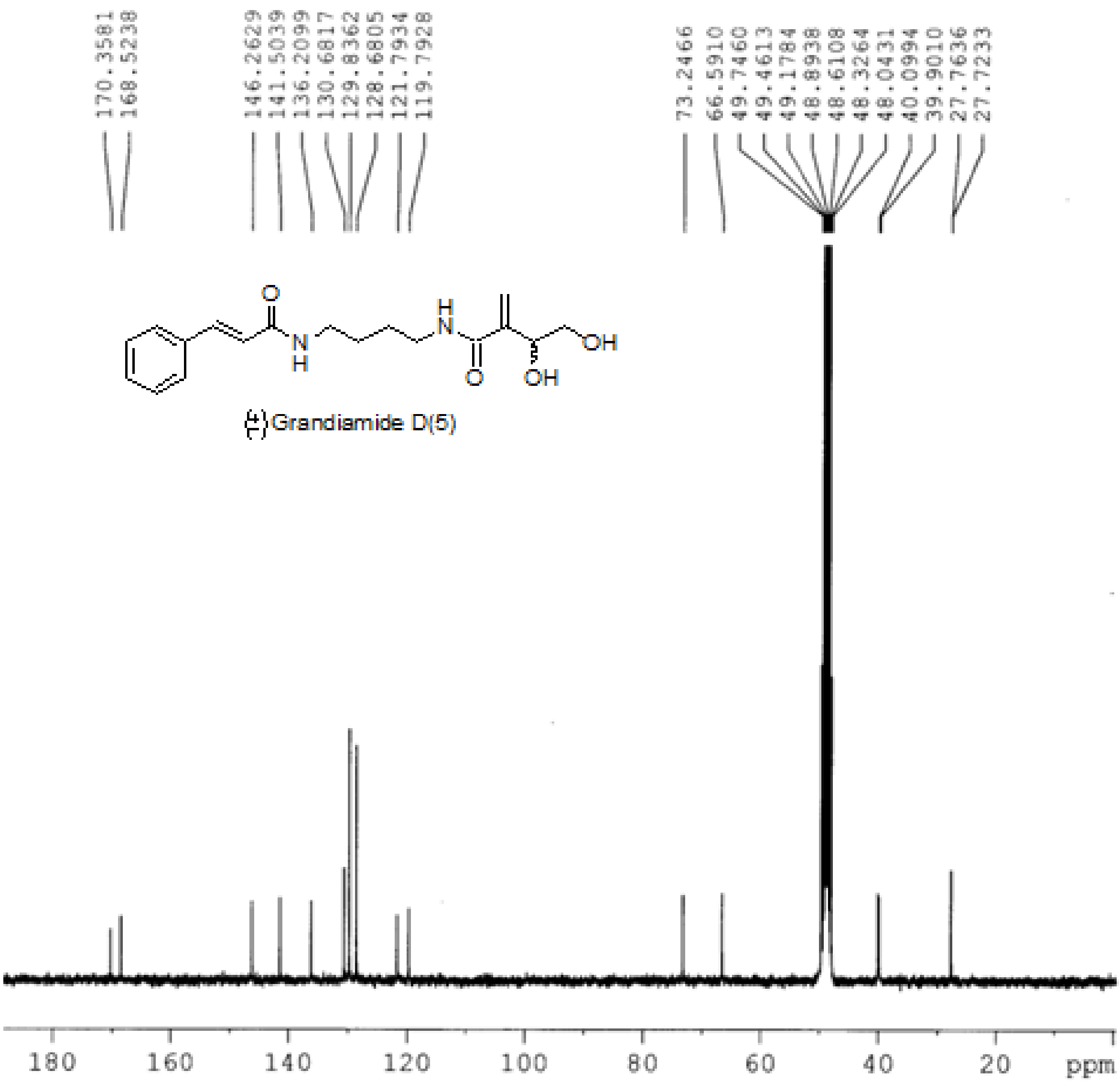
170.3581
168.5238
146.2629
141.5039
136.2099
130.6817
129.8362
128.6805
121.7934
119.7928
73.2466
66.5910
49.7460
49.4613
49.1784
48.8938
48.6108
48.3264
48.0431
40.0994
39.9010
27.7636
27.7233
(±) Grandiamide D(5)
180
160
140
120
100
80
60
40
20
ppm

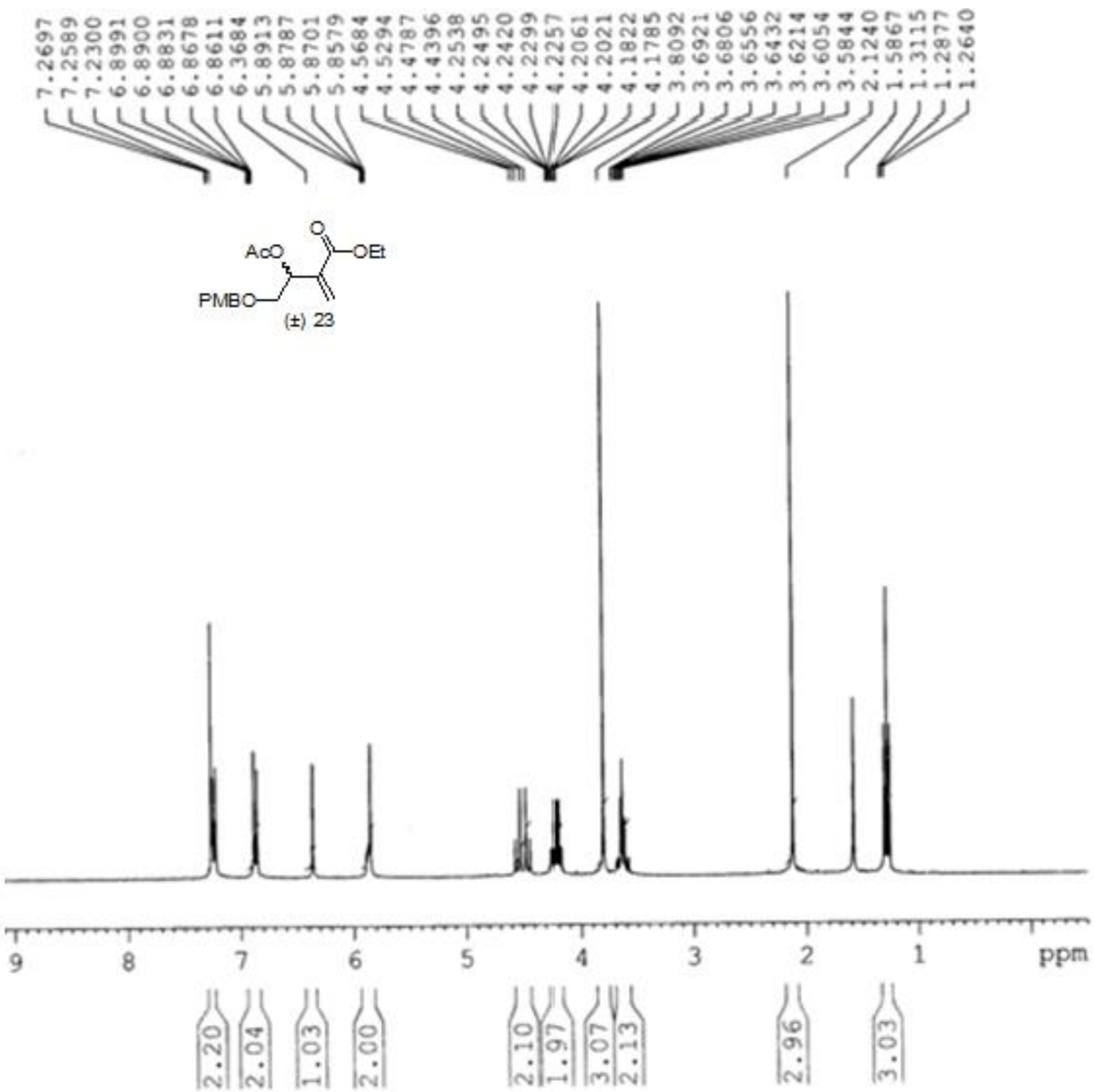

7.2697
7.2589
7.2300
6.8991
6.8900
6.8831
6.8678
6.8611
6.3684
5.8913
5.8787
5.8701
5.8579
4.5684
4.5294
4.4787
4.4396
4.2538
4.2495
4.2420
4.2299
4.2257
4.2061
4.2021
4.1822
4.1785
3.8092
3.6921
3.6806
3.6556
3.6432
3.6214
3.6054
3.5844
2.1240
1.5867
1.3115
1.2877
1.2640
AcO
OEt
PMBO
(±) 23
9
8
7
6
5
4
3
2
1
ppm
2.20
2.04
1.03
2.00
2.10
1.97
3.07
2.13
2.96
3.03

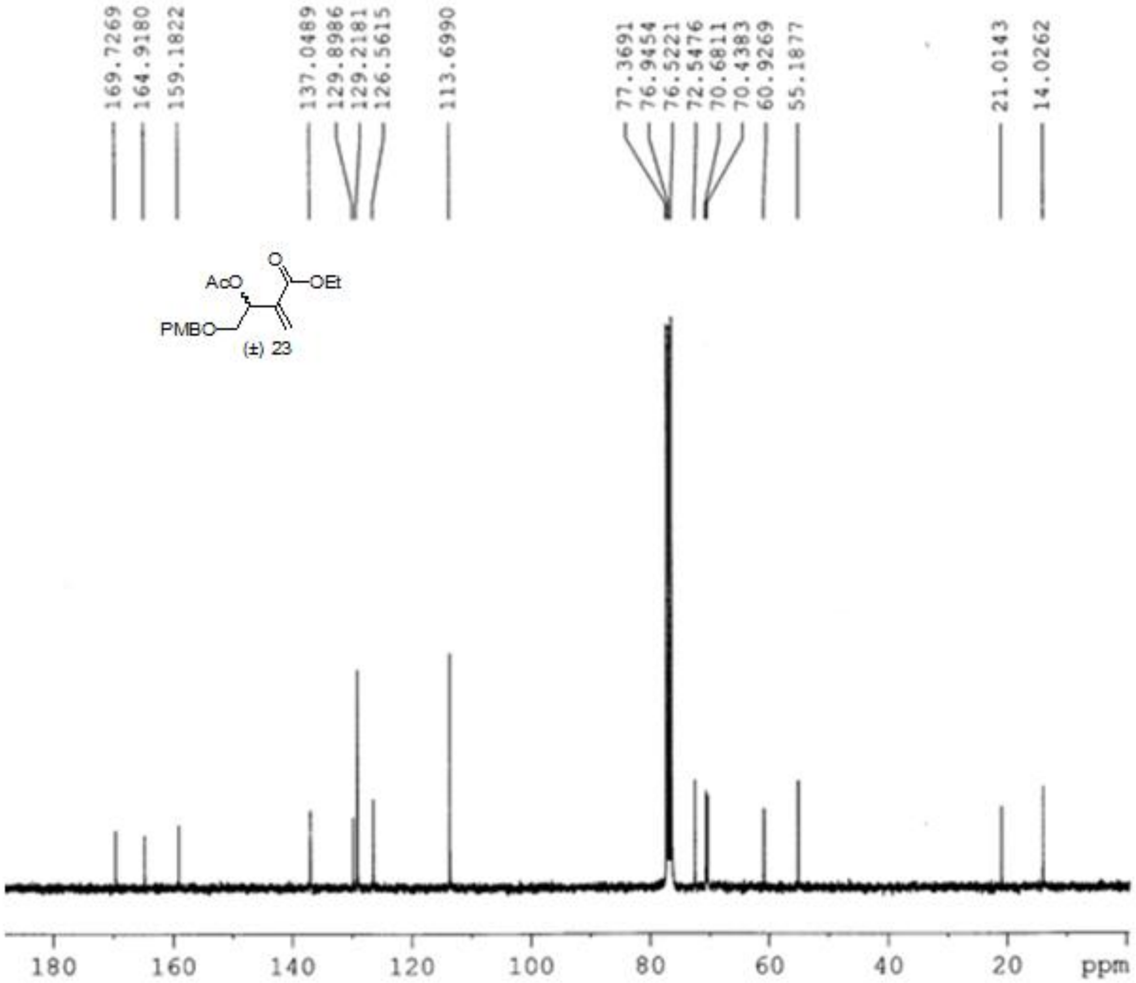
169.7269
164.9180
159.1822
137.0489
129.8986
129.2181
126.5615
113.6990
77.3691
76.9454
76.5221
72.5476
70.6811
70.4383
60.9269
55.1877
21.0143
14.0262
AcO
OEt
PMBO
(±) 23
180
160
140
120
100
80
60
40
20
ppm

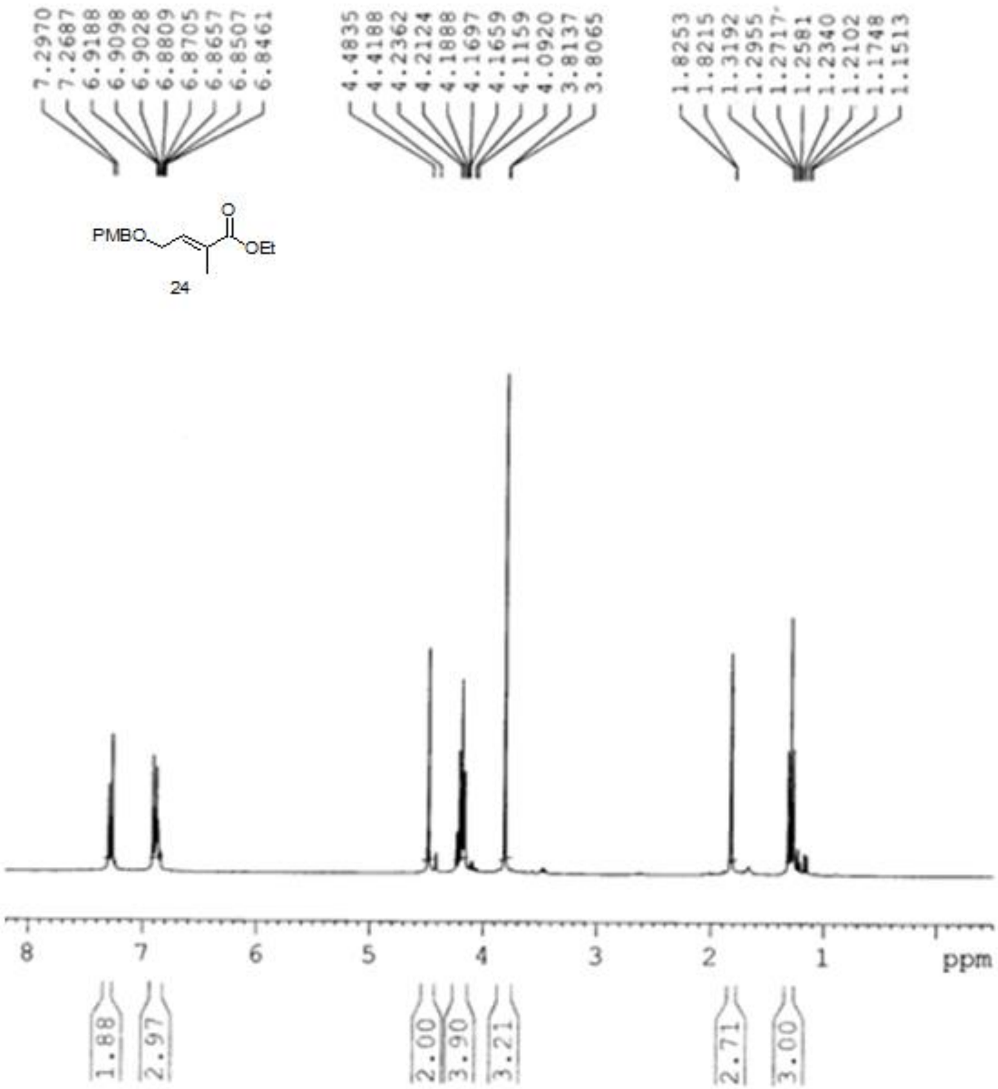

7.2970
7.2687
6.9188
6.9098
6.9028
6.8809
6.8705
6.8657
6.8507
6.8461
4.4835
4.4188
4.2362
4.2124
4.1888
4.1697
4.1659
4.1159
4.0920
3.8137
3.8065
1.8253
1.8215
1.3192
1.2955
1.2717
1.2581
1.2340
1.2102
1.1748
1.1513
PMBO
OEt
O
24
8
7
6
5
4
3
2
1
ppm
1.88
2.97
2.00
3.90
3.21
2.71
3.00

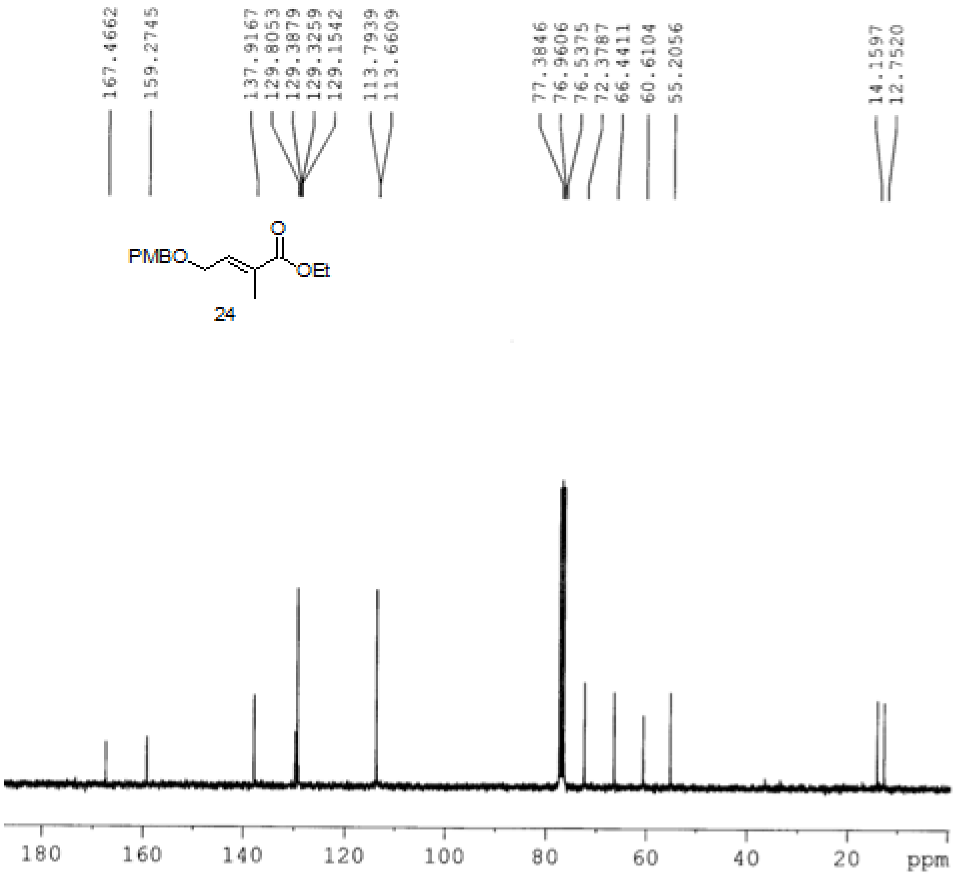
167.4662
159.2745
137.9167
129.8053
129.3879
129.3259
129.1542
113.7939
113.6609
77.3846
76.9606
76.5375
72.3787
66.4411
60.6104
55.2056
14.1597
12.7520
PMBO
OEt
O
24
180
160
140
120
100
80
60
40
20
ppm

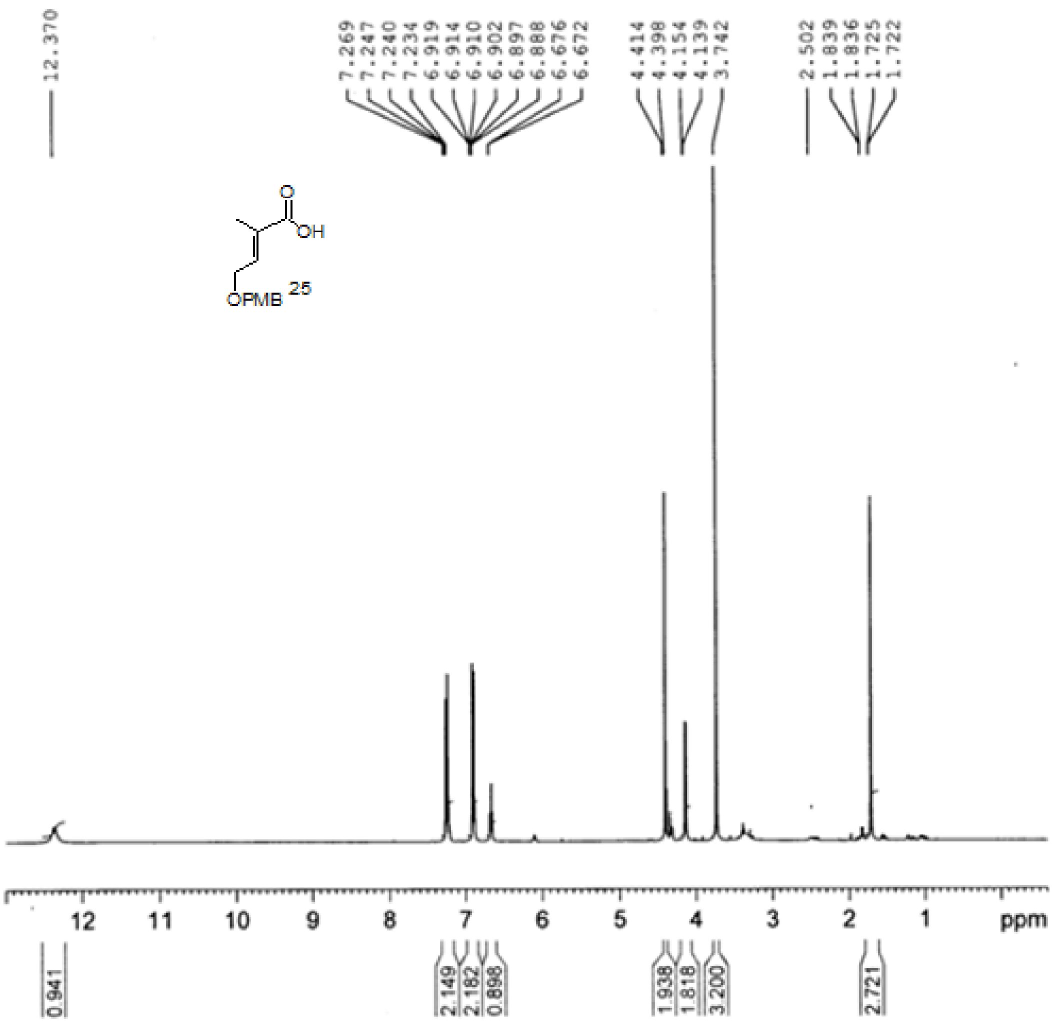
12.370
7.269
7.247
7.240
7.234
6.919
6.914
6.910
6.902
6.897
6.888
6.676
6.672
4.414
4.398
4.154
4.139
3.742
2.502
1.839
1.836
1.725
1.722
O
OH
OPMB
25
12
11
10
9
8
7
6
5
4
3
2
1
ppm
0.941
2.149
2.182
0.898
1.938
1.818
3.200
2.721

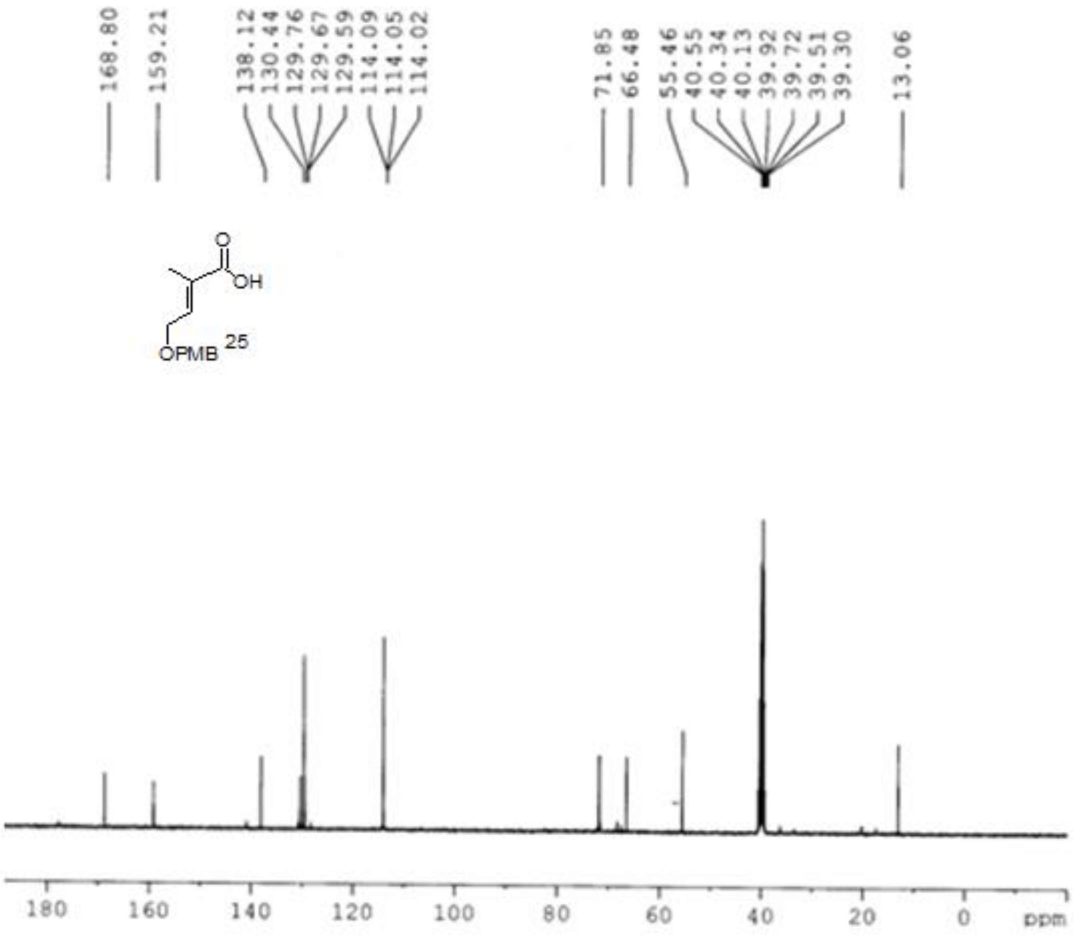
168.80
159.21
138.12
130.44
129.76
129.67
129.59
114.09
114.05
114.02
71.85
66.48
55.46
40.55
40.34
40.13
39.92
39.72
39.51
39.30
13.06
O
OH
OPMB
25
180
160
140
120
100
80
60
40
20
0
ppm

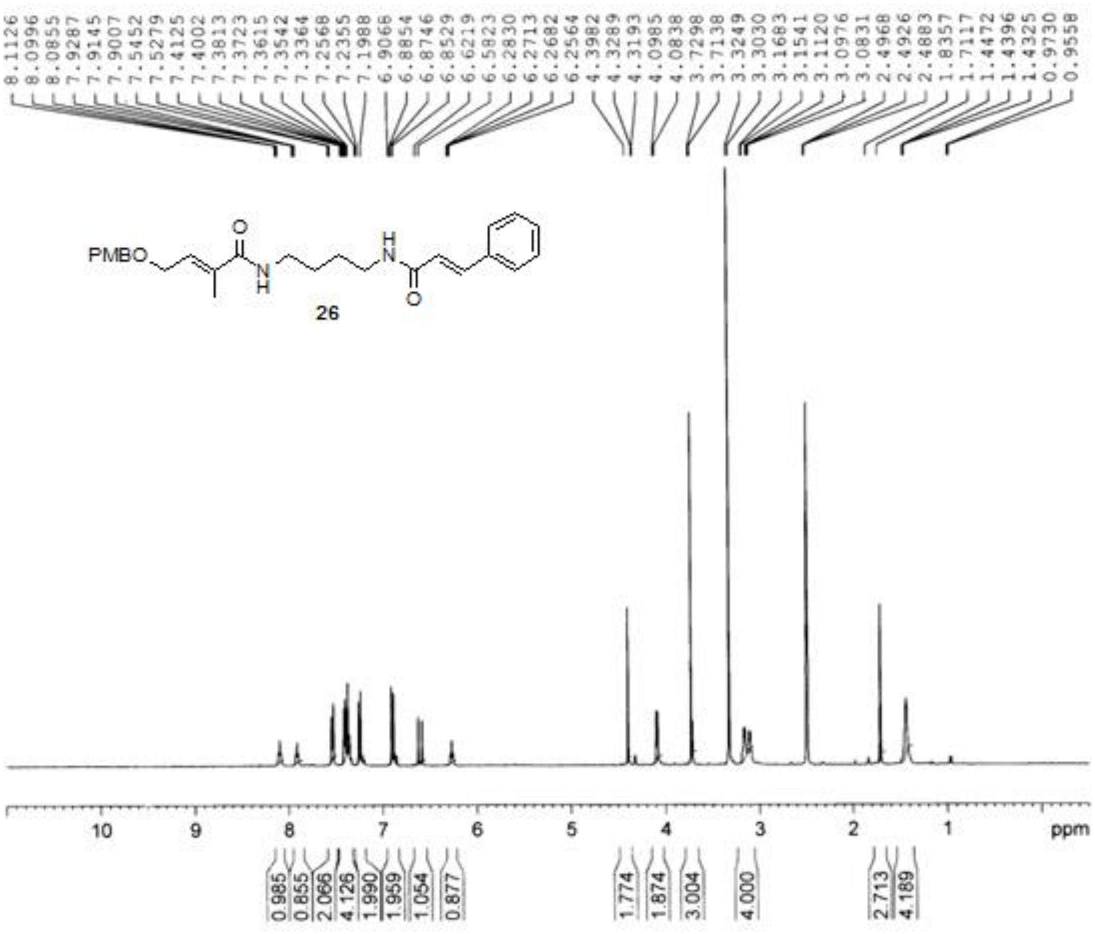

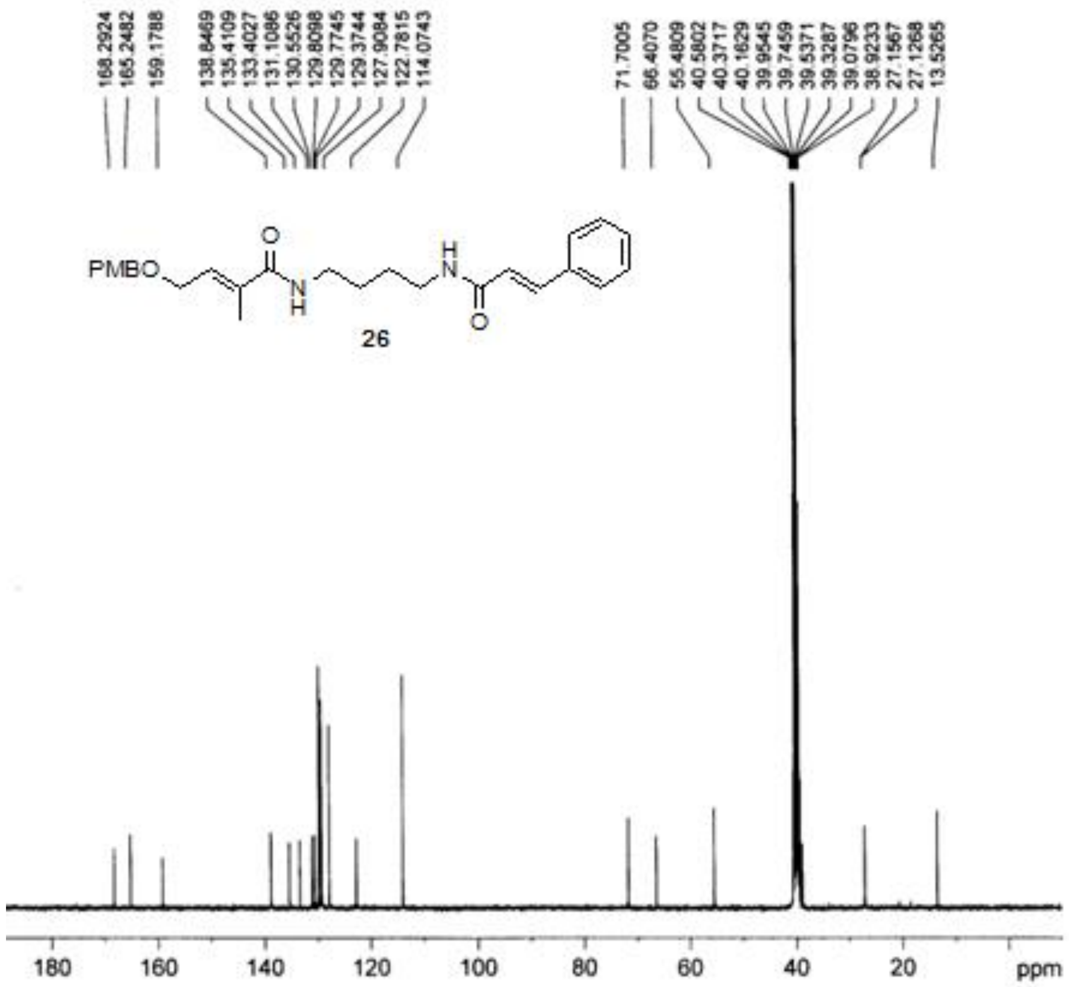

168.2924
165.2482
159.1788
138.8469
135.4109
133.4027
131.1086
130.5526
129.8098
129.7745
129.3744
127.9084
122.7815
114.0743
71.7005
66.4070
55.4809
40.5802
40.3717
40.1629
39.9545
39.7459
39.5371
39.3287
39.0796
38.9233
27.1567
27.1268
13.5265
PMBO
26
180
160
140
120
100
80
60
40
20
ppm

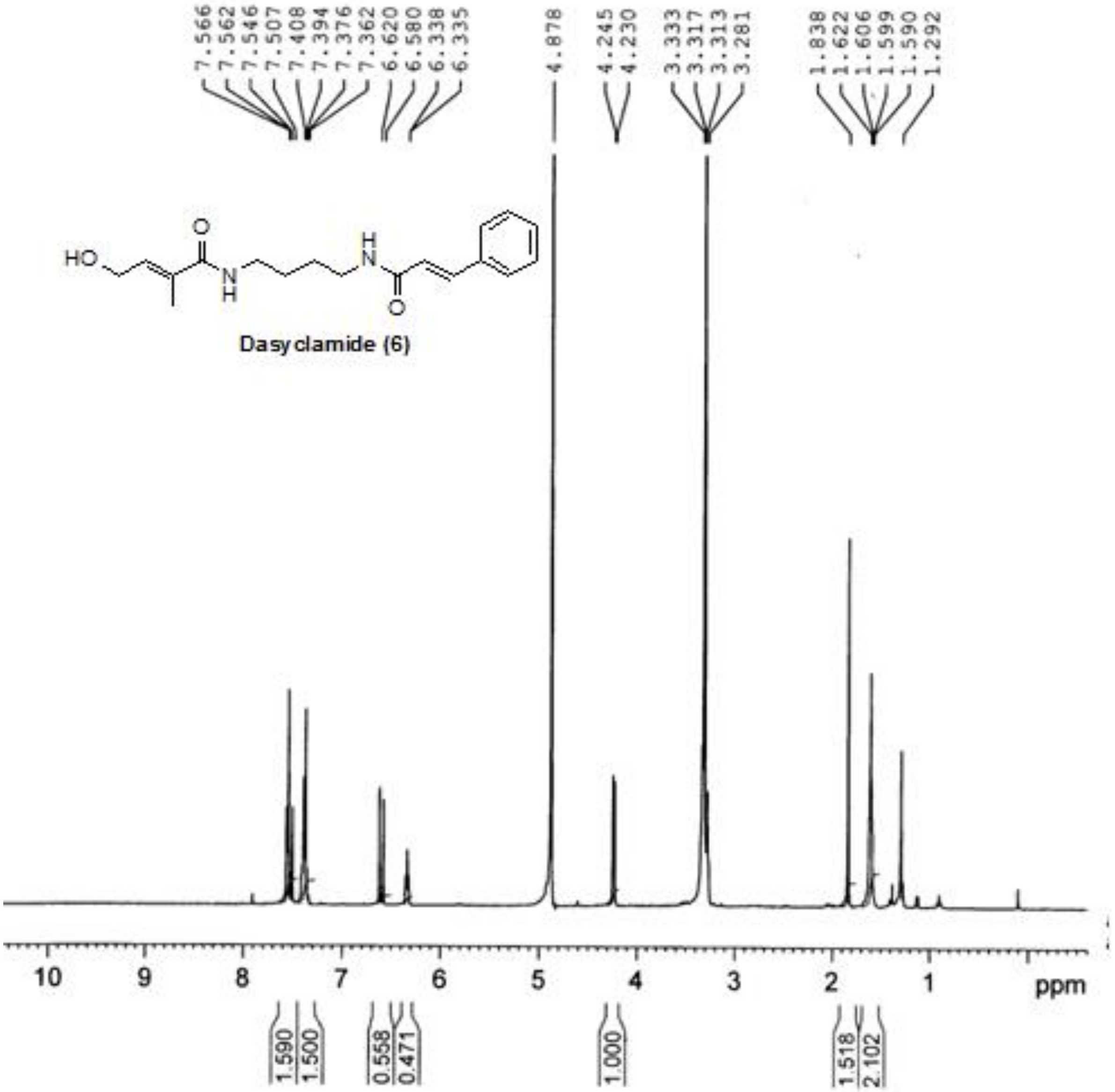
7.566
7.562
7.546
7.507
7.408
7.394
7.376
7.362
6.620
6.580
6.338
6.335
4.878
4.245
4.230
3.333
3.317
3.313
3.281
1.838
1.622
1.606
1.599
1.590
1.292
HO
O
H
N
N
H
O
Dasyclamide (6)
10
9
8
7
6
5
4
3
2
1
ppm
1.590
1.500
0.558
0.471
1.000
1.518
2.102

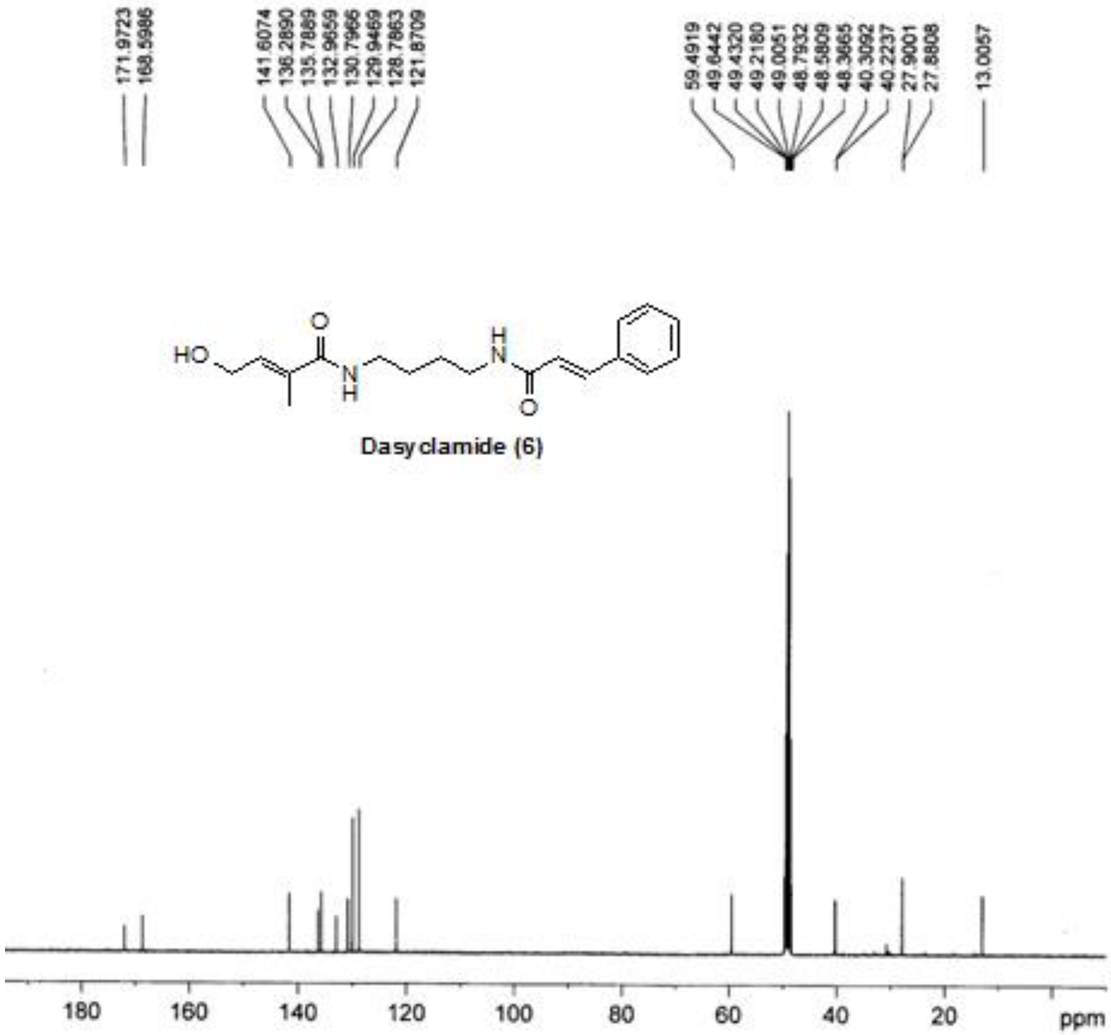

Dasyclamide (6)

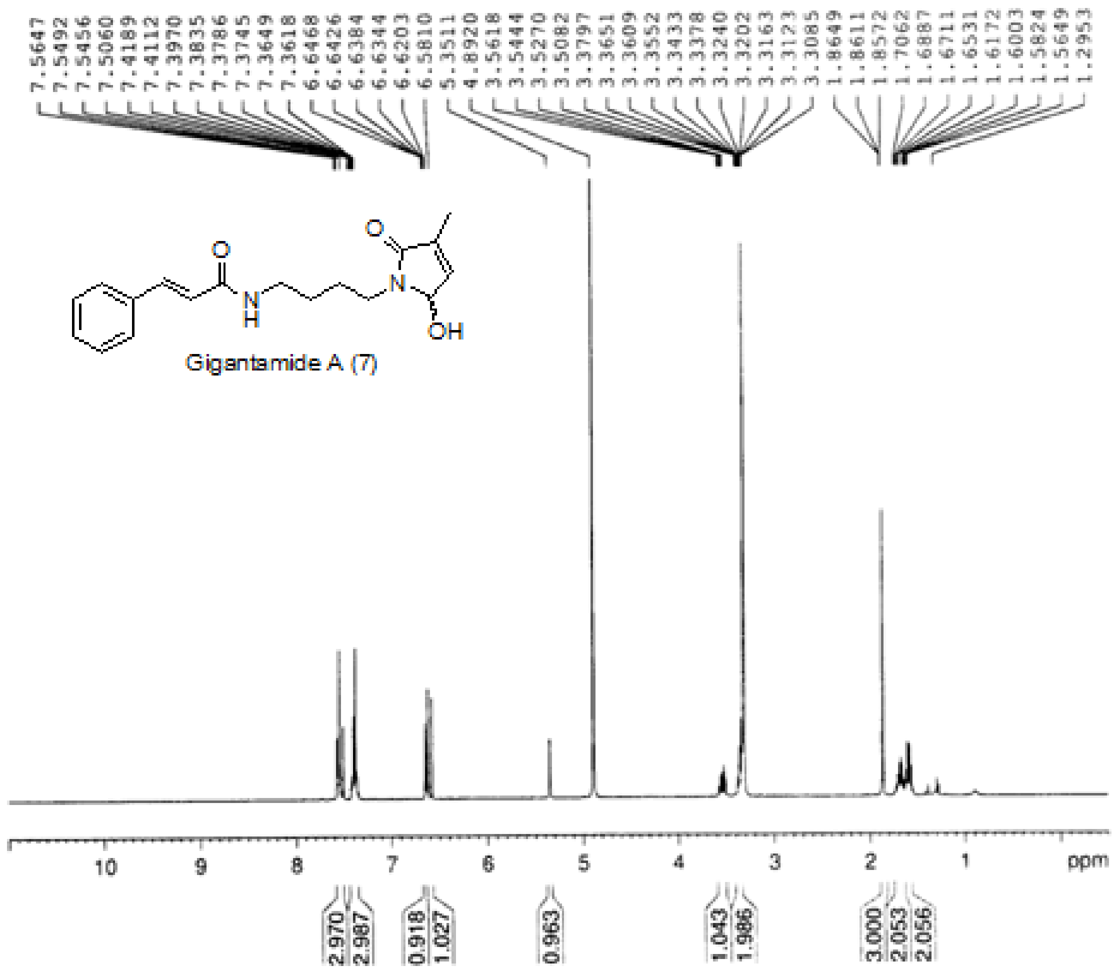
7.5647
7.5492
7.5456
7.5060
7.4189
7.4112
7.3970
7.3835
7.3786
7.3745
7.3649
7.3618
6.6468
6.6426
6.6384
6.6344
6.6203
6.5810
5.3511
4.8920
3.5618
3.5444
3.5270
3.5082
3.3797
3.3651
3.3609
3.3552
3.3433
3.3378
3.3240
3.3202
3.3163
3.3123
3.3085
1.8649
1.8611
1.8572
1.7062
1.6887
1.6711
1.6531
1.6172
1.6003
1.5824
1.5649
1.2953
O
O
N
H
N
OH
Gigantamide A (7)
10
9
8
7
6
5
4
3
2
1
ppm
2.970
2.987
0.918
1.027
0.963
1.043
1.986
3.000
2.053
2.056

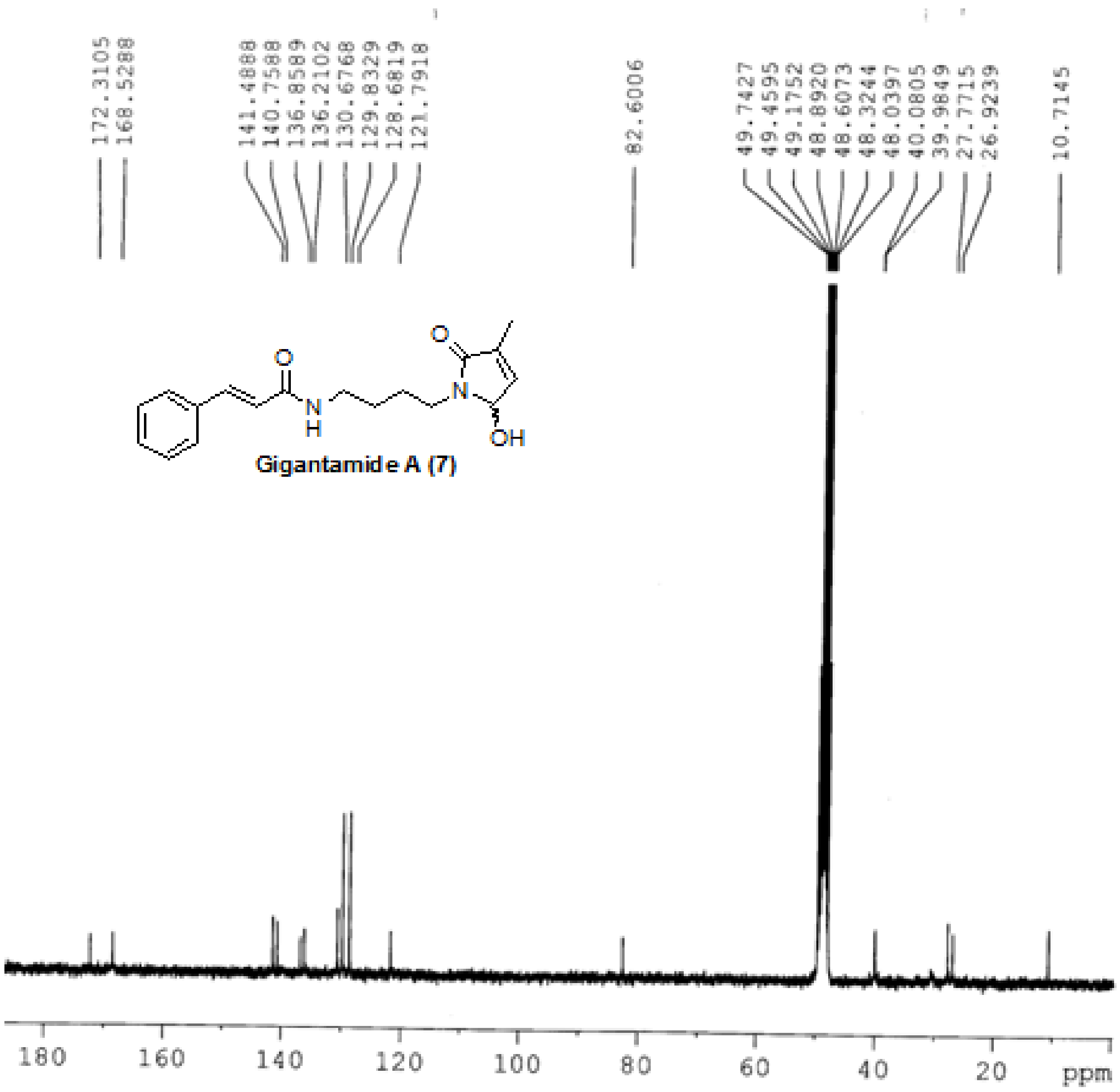
172.3105
168.5288
141.4888
140.7588
136.8589
136.2102
130.6768
129.8329
128.6819
121.7918
82.6006
49.7427
49.4595
49.1752
48.8920
48.6073
48.3244
48.0397
40.0805
39.9849
27.7715
26.9239
10.7145
N
H
O
N
OH
Gigantamide A (7)
180
160
140
120
100
80
60
40
20
ppm

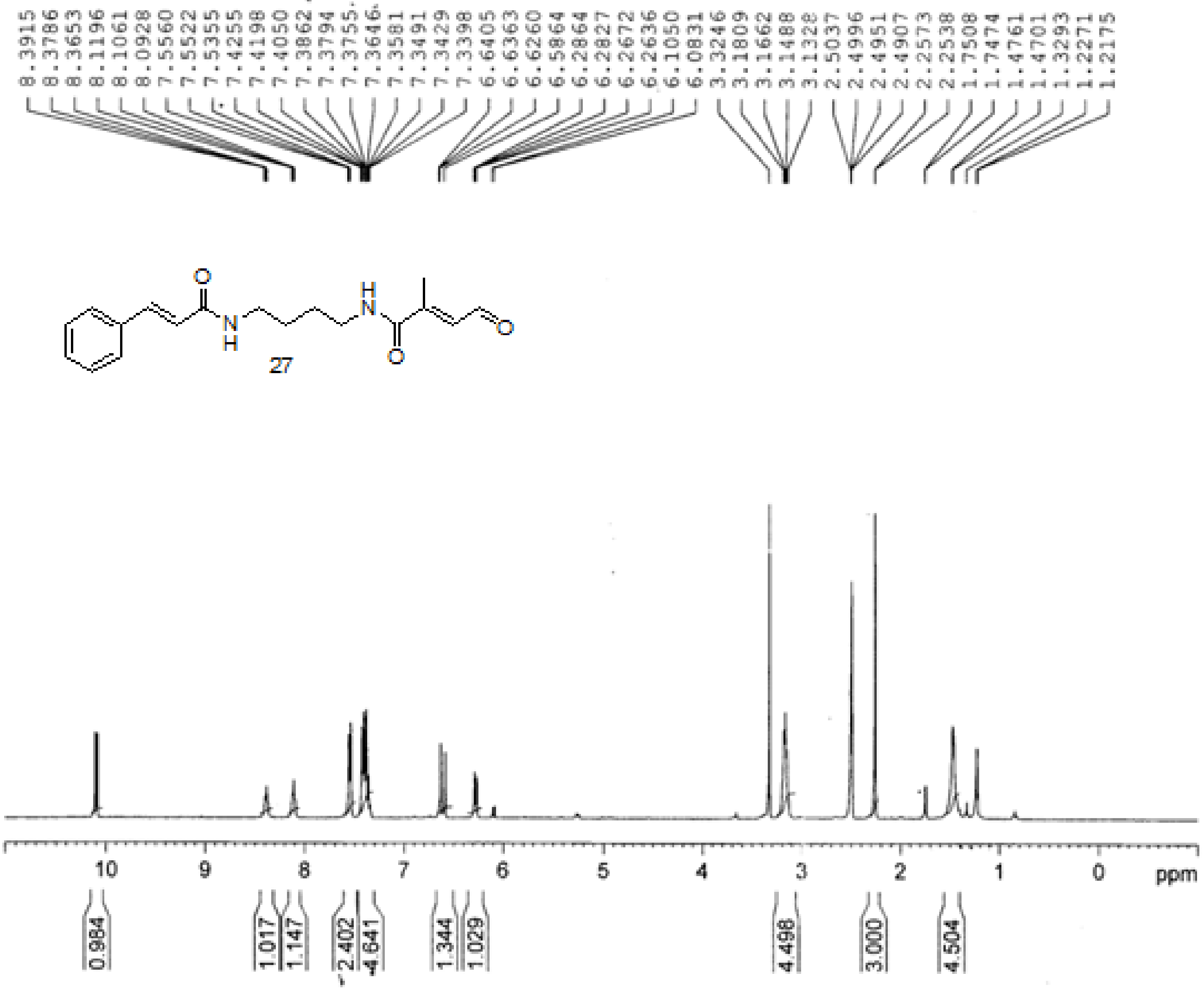
8.3915
8.3786
8.3653
8.1196
8.1061
8.0928
7.5560
7.5522
7.5355
7.4255
7.4198
7.4050
7.3862
7.3794
7.3755
7.3646
7.3581
7.3491
7.3429
7.3398
6.6405
6.6363
6.6260
6.5864
6.2864
6.2827
6.2672
6.2636
6.1050
6.0831
3.3246
3.1809
3.1662
3.1488
3.1328
2.5037
2.4996
2.4951
2.4907
2.2573
2.2538
1.7508
1.7474
1.4761
1.4701
1.3293
1.2271
1.2175
27
10
9
8
7
6
5
4
3
2
1
0
ppm
0.984
1.017
1.147
2.402
4.641
1.344
1.029
4.498
3.000
4.504

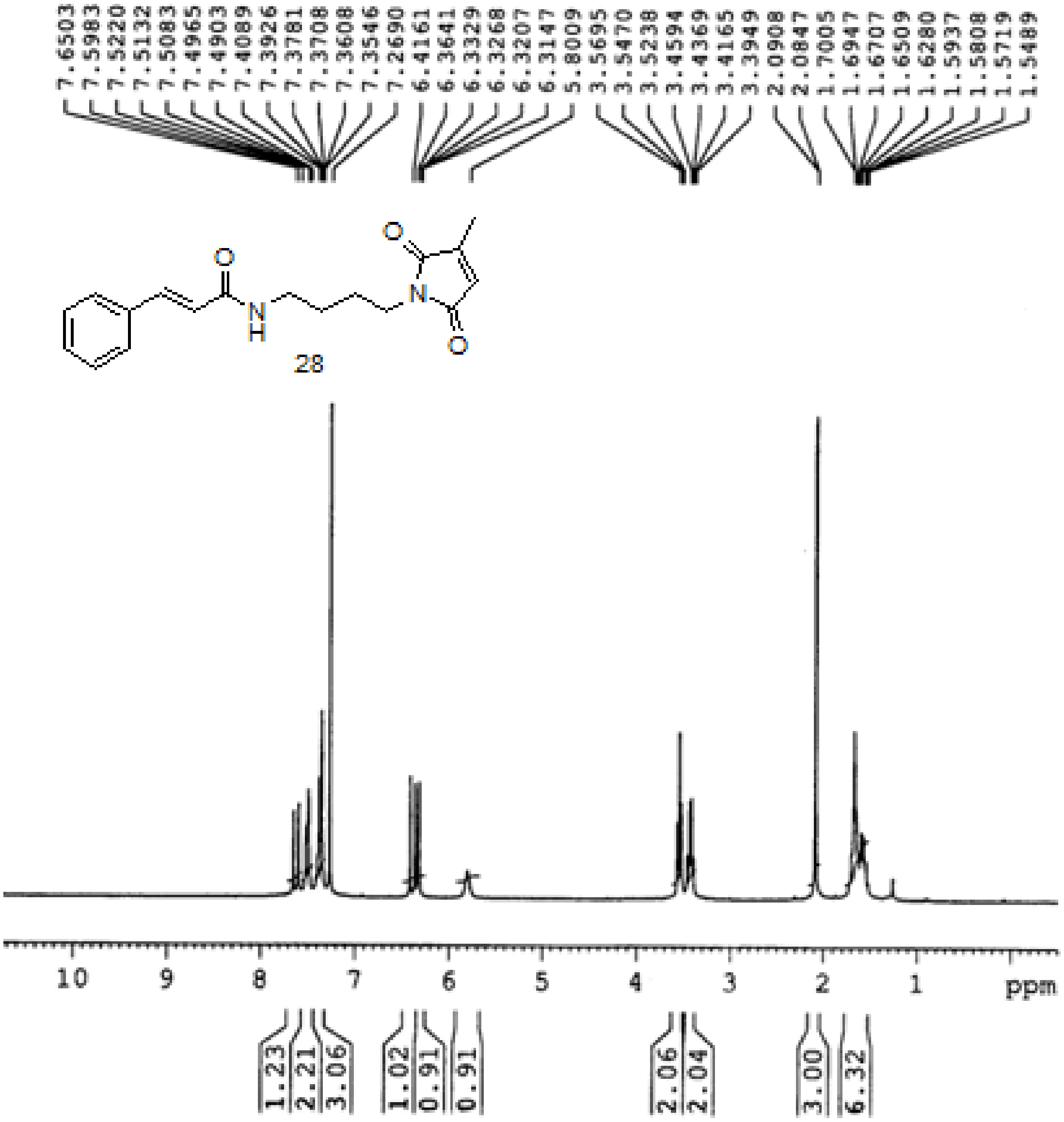
7.6503
7.5983
7.5220
7.5132
7.5083
7.4965
7.4903
7.4089
7.3926
7.3781
7.3708
7.3608
7.3546
7.2690
6.4161
6.3641
6.3329
6.3268
6.3207
6.3147
5.8009
3.5695
3.5470
3.5238
3.4594
3.4369
3.4165
3.3949
2.0908
2.0847
1.7005
1.6947
1.6707
1.6509
1.6280
1.5937
1.5808
1.5719
1.5489
O
N
H
O
N
O
28
10
9
8
7
6
5
4
3
2
1
ppm
1.23
2.21
3.06
1.02
0.91
0.91
2.06
2.04
3.00
6.32

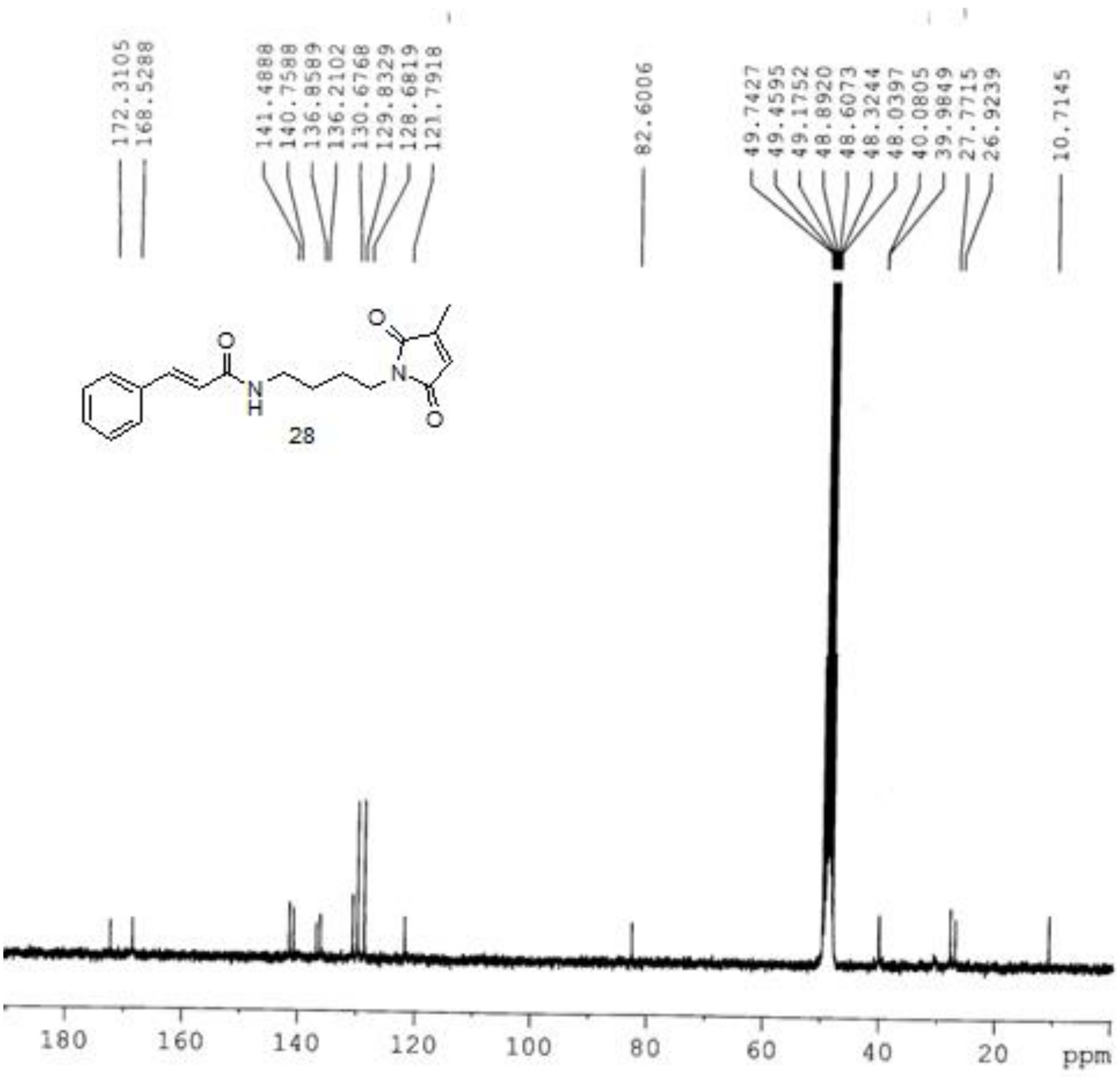
172.3105
168.5288
141.4888
140.7588
136.8589
136.2102
130.6768
129.8329
128.6819
121.7918
82.6006
49.7427
49.4595
49.1752
48.8920
48.6073
48.3244
48.0397
40.0805
39.9849
27.7715
26.9239
10.7145
28
180
160
140
120
100
80
60
40
20
ppm

Chiral HPLC reports

```
Method info  :  Column : Chiral pak ADH  (250 X4.6)mm,
                Mobile Phase : 0.%TFA in Hexane :Ethanol (80:20)
                Flow :1.0 ml\min
```

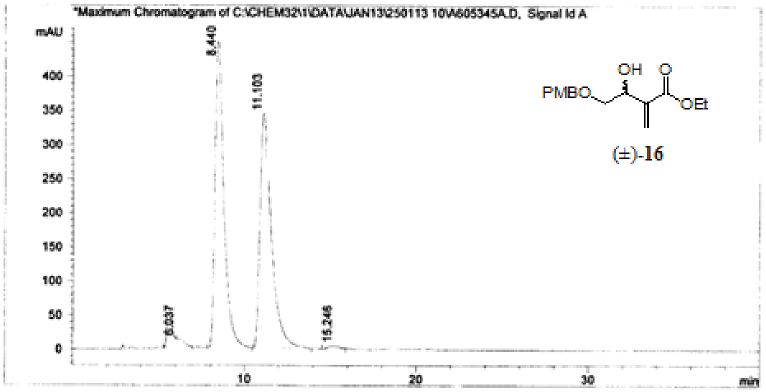

Peak No	RT min	Area	Area %
1	5.590	1259.885	3.715
2	6.037	380.721	1.123
3	8.440	16059.144	47.349
4	11.103	16018.755	47.230
5	15.246	198.253	0.585

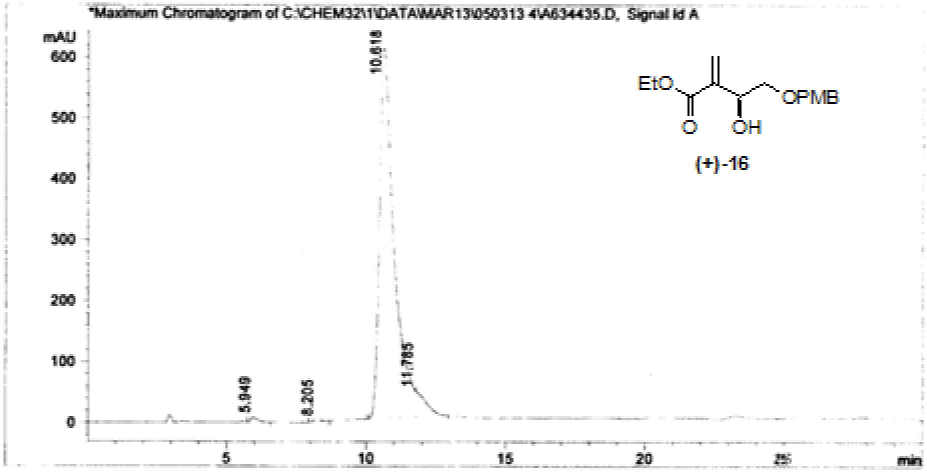

Peak No	RT min	Area	Area %
1	5.949	135.784	0.587
2	8.205	64.320	0.278
3	10.618	21860.453	94.509
4	11.785	1069.912	4.626

```
Method info  :  Column : Chiralpak ADH (250 X4.6)mm,5um
                Mobile Phase :  0.1%TFA IN Hexane :Ethanol(85:15)
                Flow :1.0 ml\min
```

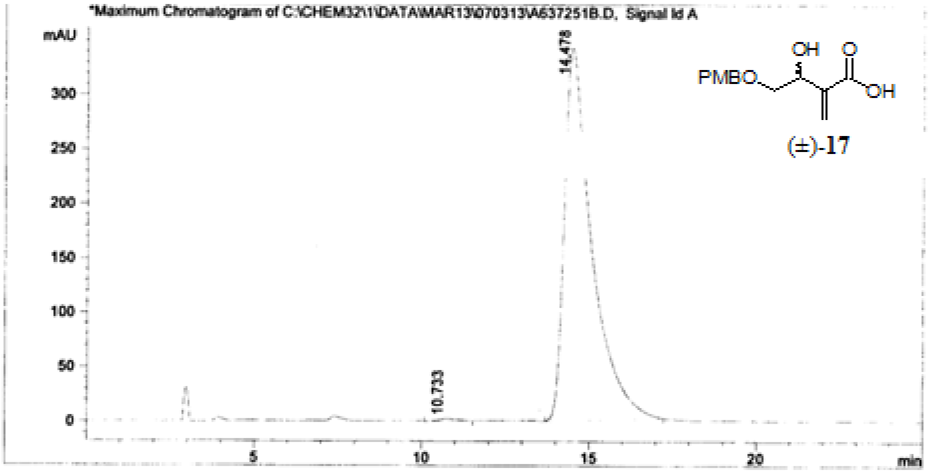

Peak No	RT min	Area	Area %
1	10.733	63.695	0.326
2	14.478	19470.041	99.674

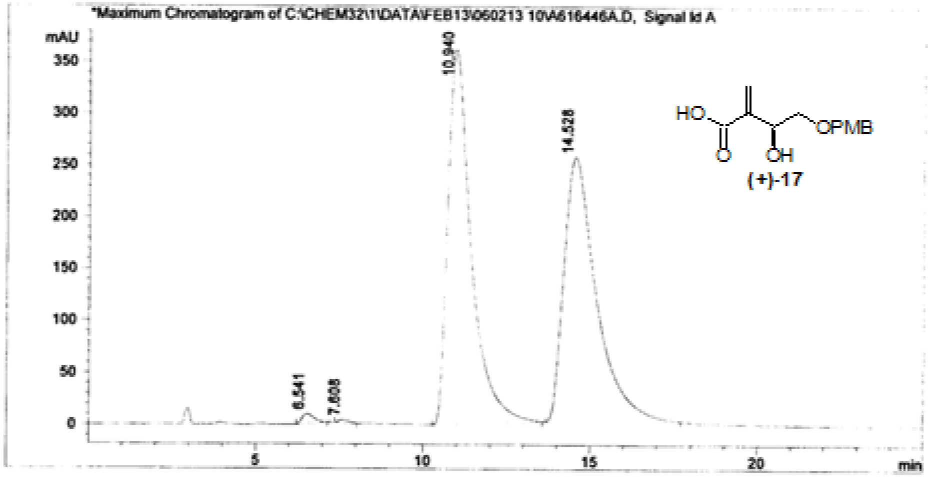

Peak No	RT min	Area	Area %
1	6.541	227.685	0.647
2	7.608	66.632	0.189
3	10.940	17666.137	50.219
4	14.528	17217.391	48.944

Method information : Column: Chiral pak IC250 X 4.6)mm 5u
Mobile Phase 'A' : Hexane : Ethanol (70:30)
Flow :1.0ml/min

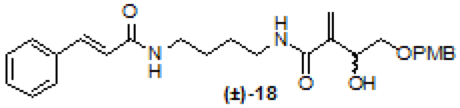

(±)-18

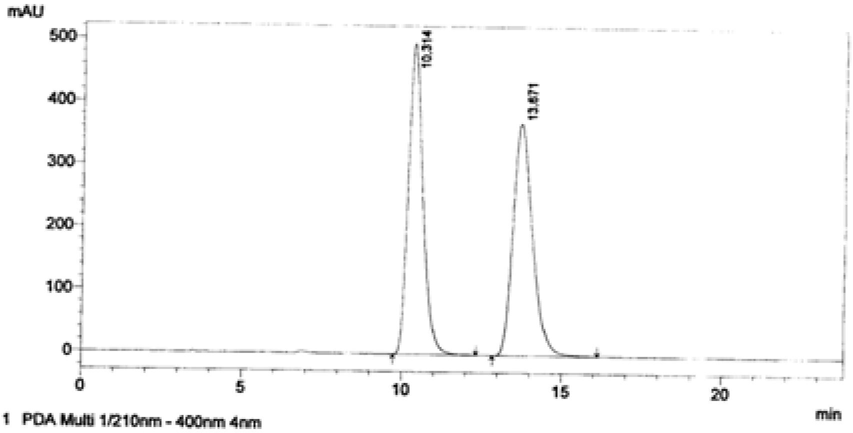

1 PDA Multi 1/210nm - 400nm 4nm

PeakTable

PDA Ch1 210nm - 400nm 4nm

Peak#	Ret. Time	Area	Area %
1	10.314	15764351	50.122
2	13.671	15687718	49.878
Total		31452069	100.000

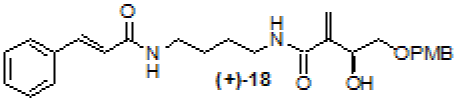

(+)-18

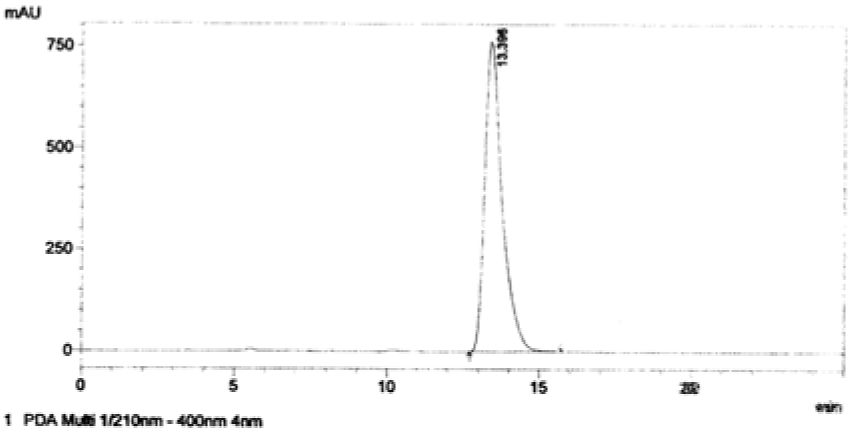

1 PDA Multi 1/210nm - 400nm 4nm

PeakTable

PDA Ch1 210nm - 400nm 4nm

Peak#	Ret. Time	Area	Area %
1	13.396	30391490	100.000
Total		30391490	100.000

```
Method info  :  Column : Chiralpak IC (250 X4.6)mm,
                Mobile Phase :  Hexane :Ethanol(30:70)
                Flow :1.0 ml\min
```

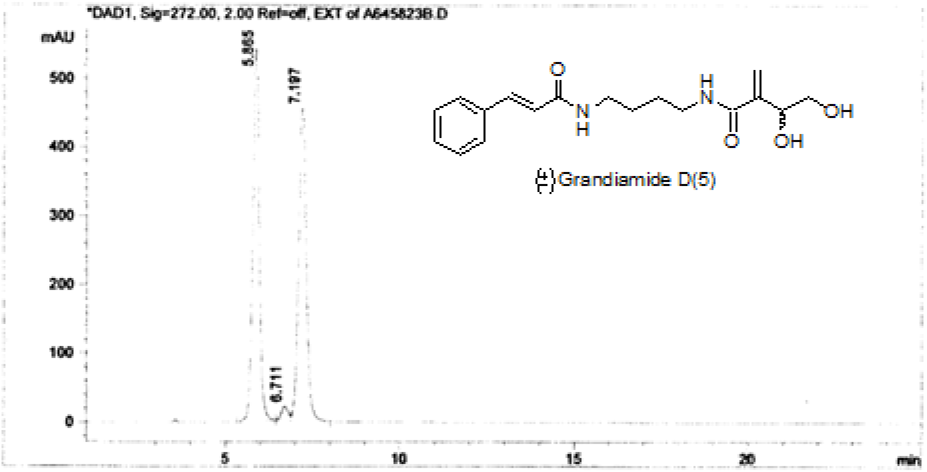

Peak No	RT min	Area	Area %
1	5.865	6958.548	48.993
2	6.711	263.666	1.856
3	7.197	6980.845	49.150

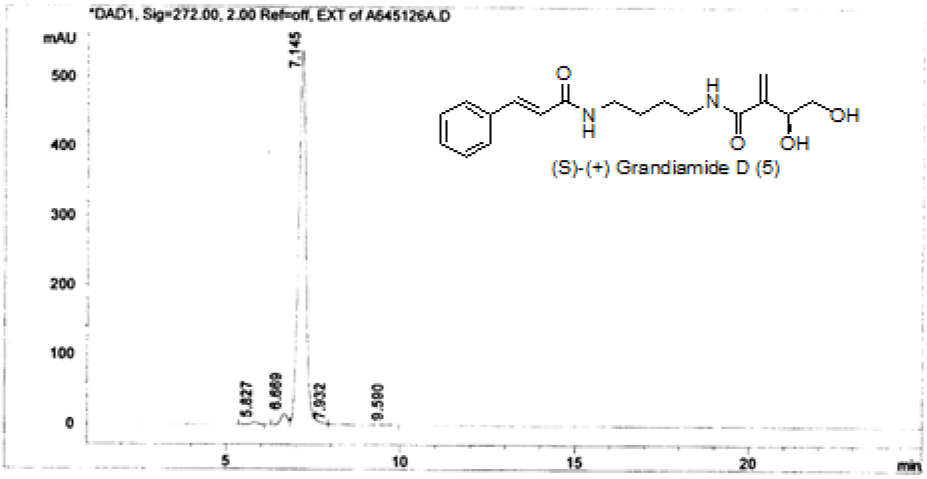

Peak No	RT min	Area	Area %
1	5.827	55.701	0.678
2	6.669	200.197	2.436
3	7.145	7907.957	96.243
4	7.932	39.442	0.480
5	9.590	13.352	0.163

Method information :ChiralCEL ODH(250 X 4.6)mm 5um,
Mobile Phase : 0.1%TFA IN Hexane:ethanol (90:10)
Flow : 1.0ml/min

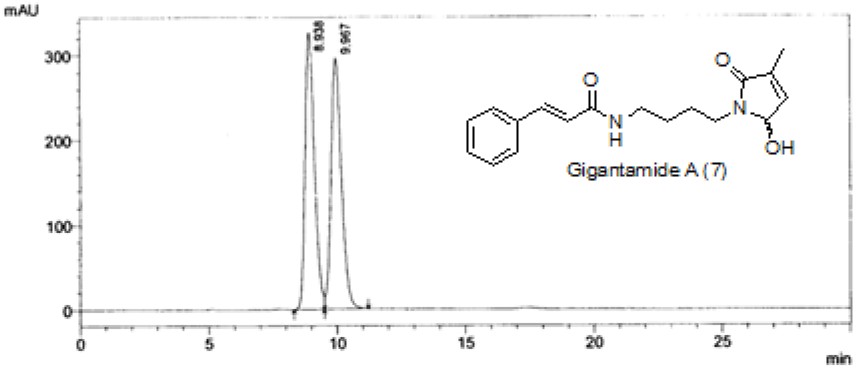

1 PDA Multi 1/270nm 4nm

PeakTable

PDA Ch1 270nm 4nm

Peak#	Ret. Time	Area	Area %
1	8.938	8278650	49.587
2	9.967	8416597	50.413
Total		16695247	100.000
